# Assessment of Individual Exposure to Multiple Pollutants (Noise, Particulate Matter, and Extremely Low-Frequency Magnetic Fields) Related to Daily Life Microenvironments in the Brussels Capital Region: Protocol for a Cross-Sectional Study

**DOI:** 10.2196/69407

**Published:** 2025-07-03

**Authors:** Agathe O Salmon, Maryse Ledent, Eva M De Clercq, Bram Vanhoutte, Catherine Bouland

**Affiliations:** 1 Environmental and Occupational Health Research Centre School of Public Health Université Libre de Bruxelles Brussels Belgium; 2 Department of Chemical and Physical Health Risks Sciensano Brussels Belgium; 3 Epidemiology and Biostatistics Research Centre School of Public Health Université Libre de Bruxelles Brussels Belgium

**Keywords:** personal exposure, multiple exposures, noise, particulate matter, PM, extremely low-frequency magnetic fields, ELF-MF, pollutants, microenvironment

## Abstract

**Background:**

Environmental factors are responsible for 13% of annual deaths in Europe. Citizens are constantly exposed to a variety of environmental factors, such as noise, air pollutants, and magnetic fields (MFs), which may interact with one another. To study multiple-pollutant exposures simultaneously, data on individual citizens, collected using portable measuring devices, provide a high level of detail for exposure characterization.

**Objective:**

The aims of this study are to (1) assess the exposure of urban citizens to multiple pollutants (noise, particulate matter [PM], and extremely low-frequency magnetic fields [ELF-MFs]) on a normal weekday, (2) estimate the contribution of each main daily life microenvironment in the multiple-pollutant exposure, and (3) estimate the role of measured exposure in the assessment of perceived personal exposure.

**Methods:**

We collected the exposure levels of 490 individuals to multiple pollutants: PM, ELF-MFs, and noise levels. We used 3 devices per participant (Airbeam 2, EMDEX II or EMDEX Lite, and a smartphone with the Aircasting app for PM, ELF-MFs, and noise, respectively). Participants wore them for 24 hours on a normal weekday. In parallel, they filled out a microenvironment diary and a questionnaire focusing on socioeconomic data, lifestyle, and perceived exposures. The analysis will first describe the exposures as daily averages and aggregated by microenvironment. Several analyses will be conducted: (1) an estimation of the contribution of each microenvironment in the exposure levels of the 3 pollutants studied, (2) a linear mixed model (for each pollutant) to explain the measured levels of exposure, and (3) linear regression to assess the contribution of the measured personal exposure in self-reported perceived exposures.

**Results:**

Data collection was carried out from October 2020 to August 2022, with 490 individuals taking part. The databases have been gathered and cleaned. Future work will focus on data analysis.

**Conclusions:**

The collected data will allow us to describe the daily multiple-pollutant exposures faced by individuals within the general population and to characterize the main microenvironments of their daily lives according to multiple-pollutant exposures. This will help identify precise microenvironments to be targeted in policies aiming to reduce exposure to pollution. Because the sampling method is not probabilistic, it is not expected to be representative of the population of the Brussels Capital Region, but it will provide a first step in the understanding of multiple-pollutant exposures faced by individual citizens.

**International Registered Report Identifier (IRRID):**

DERR1-10.2196/69407

## Introduction

### Background

According to the World Health Organization (WHO), 13% of deaths in the European Union (EU) in 2012 (last update) were linked to environmental factors [1]. This represents 630,000 deaths each year, of which 90% are due to noncommunicable diseases, the main ones being cancer, ischemic heart disease, chronic obstructive pulmonary disease (COPD), and stroke. In addition to deaths, WHO estimates that 20 million healthy life-years are lost worldwide each year due to environmental factors [1]. Since populations are generally exposed to multiple pollutants at once, the European Environment Agency underlines the importance of studying multiple-pollutant exposures and their impacts on health [2]. The 2 main environmental factors negatively impacting human health are air pollution and noise [2].

The main air pollutants are particulate matter (PM), ozone (O_3_), and nitrogen dioxide (NO_2_). Among these, PM_2.5_ (aerodynamic diameter<2.5 µm) is the largest contributor, with 379,000 of the 400,000 premature deaths attributed to air pollution in the EU-28 (European Union with 28 member states) in 2018 [2]. This is despite a steady decline in PM concentrations in Europe since 1990 [2]. Between 2016 and 2018, more than 74% of Europe’s urban population was exposed to PM_2.5_ levels exceeding the WHO threshold. PM affects the human body by causing respiratory problems, cardiovascular diseases, COPD, and even reproductive issues. Concerning indoor air quality, PM is also the first factor responsible for indoor pollutant–related diseases [2].

After air pollution, noise is the second-most important contributor to the environmental burden of disease in Europe [2]. Noise can affect human health either by damaging the auditory system (eg, hearing loss) or by inducing physiological stress (eg, sleep disturbance, impaired concentration with impacts on cognitive development, and metabolic effects potentially leading to cardiovascular diseases) [3,4]. In Europe, the main source of environmental noise is road traffic. Hänninen et al [5] estimated that 400-1500 disability-adjusted life-years (DALYs = years of life with disability + years of life lost) per million people are attributable to traffic-related noise. For example, Basner et al [4] reported that an increase of 10 dB (in LAeq, a weighted equivalent continuous sound pressure level) leads to a 7% increase in hypertension, a 15% increase in strokes, and a 17% increase in myocardial infections.

Extremely low-frequency magnetic fields (ELF-MFs) are fields produced by the movement of electrically charged particles. The “extremely low” range includes 50-60 Hz fields, which are the frequency generated by electricity distribution around the world. These fields, at chronic exposures of 0.3-0.4 µT, have in some studies been associated with an increased risk of childhood leukemia [6-9] and have therefore been classified as “possibly carcinogenic to humans” by the International Agency for Research on Cancer (IARC) [10]. To date, no causal relationship has been concluded, no action mechanism has been identified nor explained, and the Scientific Committee on Health, Environmental and Emerging Risks (SCHEER) 2024 report [11] concludes that evidence of this potential association is weak. ELF-MFs are also suspected to be linked to the development of neurodegenerative diseases, such as Alzheimer’s disease or amyotrophic lateral sclerosis (ALS) [12-16], but results are too mitigated and the evidence too weak to conclude a causal association [11,17]. Despite the absence of causal pathways, exposure to ELF-MFs and their potential health effects remain relevant due to an ongoing societal debate on perceived health risks.

Urban citizens are continuously and simultaneously exposed to a wide variety of environmental factors, such as noise, air pollution, and magnetic fields (MFs), that originate from different sources and can enter the human organism in different ways. Little is known about how simultaneous coexposure to multiple types of pollutants leads to interactive, or cocktail, effects. It is hypothesized that the interaction of different exposures may lead to antagonism, synergy, or the cumulation of effects [18]. For example, it has been observed that combined exposure to air pollution and high temperatures leads to higher levels of morbidity and mortality [2].

Urban populations are particularly affected by simultaneous exposures to noise and air pollution. Indeed, road traffic, denser in urban areas, is the main source of both poor air quality and high noise levels [2]. Moreover, both air quality and noise affect similar systems in the human body, such as the cardiovascular and endocrine systems [2].

In 2015, the Scientific Committee on Emerging and Newly Identified Health Risks (SCENIHR) underlined the possible role that ELF-MFs may play in interaction with other exposures [19]. Indeed, in the presence of 50 Hz MFs from high-voltage power lines, PM gets electrically charged and becomes “corona ions” potentially more dangerous to human health than nonelectrically charged PM [20]. Moreover, PM is suspected to be associated with similar kinds of diseases than ELF-MFs are, such as neurodegenerative diseases [21,22] or childhood leukemia [23,24].

Most studies on exposures to environmental pollutants (air and noise) use data monitored from fixed stations. They provide average estimates of exposure at the levels of geographical areas (eg, neighborhoods) through extrapolation of measurements at these locations. Although this makes it possible to study potential links between health and exposures via ecological studies, the modeled data reduce the real heterogeneity of individual exposures in each area. Several authors [18,25-27] have pointed out that this results in reduced statistical power and increased measurement error.

Collecting exposure data at the level of individual citizens has several advantages. It provides information about exposure levels, detailed to whether the participant is indoors or outdoors, indoors being where people spend up to 90% of the time [2,28]. It also provides insights into exposure at the level of microenvironments (eg, kitchens, living rooms, cars, parks), as has been conducted for air pollution [25,28-30], MFs [31], and noise levels [32]. This allows us to relate exposures to the personal profile of activities of the exposed individuals. As observed by Ma et al [32], the means of exposure (in their case to noise) can be different between citizens from the same neighborhood. Buonanno et al [29] found that women in their sample were more exposed to ultrafine particles than men because they spent more time in kitchens, whereas men were most exposed while in public transport. In addition, this level of detail helps identify potential factors associated with exposure. Sivanantham et al [33] related the multiple-pollutant exposure patterns of different schools to different characteristics, such as the age of the buildings, cleaning habits, or ventilation habits. Finally, individual exposure measurements usually have a higher frequency of measurement (eg, every minute or second), whereas fixed monitors usually measure only once an hour [34].

A literature review on exposure to environmental factors during travel by Poom et al [34] found that 60% of the studies included in their review were published after 2015 and that 65% of those studies used portable sensors to collect data. This shows a growing interest in environmental exposures and in collecting data at the individual level using portable sensors.

However, there are several limitations to individual data that need to be considered. The main challenge is that collecting samples that are representative of the population is nearly impossible. Indeed, participation is voluntary, and as pointed out by Nieuwenhuijsen et al [35], only highly motivated people will agree to wear devices for several hours or days. Canha et al [28] also remind us of the importance of using reliable devices validated by comparisons with reference measurement tools. In the end, despite their imperfections, these methods allow us to improve decision-making, while acknowledging inaccuracies and uncertainties [36].

This data collection at the individual and microenvironmental levels will be insightful for further studies on the impacts of multiple-pollutant exposures on human health, especially given the growing interest in studying exposures to pollution as they occur in real life, with multiple-pollutant exposures that occur simultaneously.

### Objectives

ExpoHealth-1 is an observational study collecting data on exposure to multiple pollutants (PM, noise, ELF-MFs) at the individual level for 490 inhabitants of the Brussels Capital Region. To the best of our knowledge, this is the first study of its kind in the region. Our objectives are to (1) assess daily exposures to PM, noise, and ELF-MFs at the individual level for a sample of inhabitants of the Brussels Capital Region, (2) assess the levels of exposure to these 3 pollutants in the main microenvironments encountered on a normal weekday and evaluate the contribution of each microenvironment in the trends of exposure measured, and (3) assess the potential role of real exposure in the reporting of perceived exposure. To this end, we collected (1) individual-level exposure data for PM, noise, and ELF-MFs over 24 hours distributed over 2 normal weekdays, (2) a microenvironment diary for each participant during the 24-hour measurement period, and (3) participant information, such as gender, age, socioeconomic status, perceived health, and perceived exposure via a self-administered questionnaire.

## Methods

### Recruitment

#### Areas of Interest

**Figure 1:**
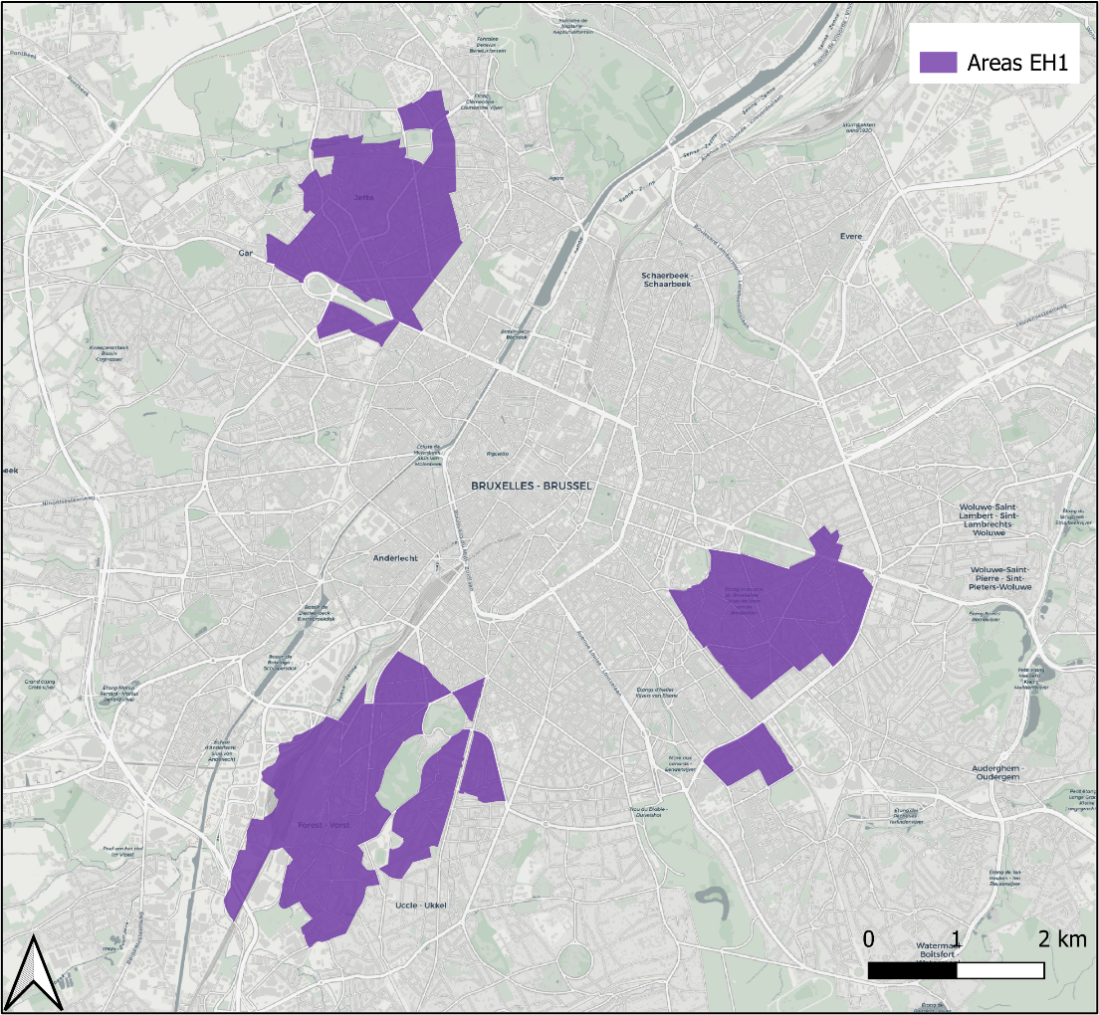
Map of the selected areas in the Brussels Capital Region.

This study is part of the Belgian Bioelectromagnetics Group (BBEMG) [37], a consortium of 7 scientific teams investigating potential health effects of exposure to ELF-MFs. For this multiple-pollutant exposure study (ExpoHealth-1), 3 geographical areas of interest have been identified in the Brussels Capital Region (Figure 1).

Statistical sectors of interest were selected based on 2 criteria. First, they had to have different expected levels of ELF-MF exposures but similar exposure levels to other environmental factors. Indeed, because environmental levels of ELF-MFs are generally low, we decided to target the potential individual exposure to this factor in the selection of areas for the study. To do so, we used data of provisional annual means of load forecast provided by the Belgian electricity system operator ELIA [38] and identified the statistical sectors potentially most and least exposed to ELF-MFs from electricity distribution. Second, each statistical sector had to include a variety of socioeconomic backgrounds to include people from different socioeconomic classes in the investigation. This choice eliminated the income extremes of statistical sectors of the city.

We included 3 environmental factors: (1) access to green spaces, (2) means of exposure to NO_2_ and PM_2.5_, and (3) variability of street exposure to ELF-MFs (as measured by a field team). We also included 3 socioeconomic factors: (1) monthly income by inhabitant, (2) employment rate, and (3) age distribution. These data (except for the ELF-MFs on-street measurements) are available in databases from the Brussels Institute of Statistics and Analysis [39]. See Multimedia Appendix 1 for more details of these factors’ levels for the selected areas.

To obtain enough volunteers, 3 groups of statistical sectors were chosen. The map (Figure 1) shows the selected areas. It was realized with QGIS 3.34.7, using a base map from Urbis [40].

The sample is nonprobabilistic as recruitment was based on volunteer participation. Invitations to participate in the data collection were made through flyers distributed in each mailbox of the selected areas. Announcements were posted on the websites of the municipal administrations and neighborhood social media groups, such as Facebook and Hoplr (see Multimedia Appendices 2 and 3). Information was also spread by word of mouth between neighbors in the selected areas.

#### Inclusion and Exclusion Criteria

Participants in the study had to live in the selected areas, be aged between 18 and 65 years, and be nonsmokers or occasional smokers. People from the same household could participate, even on the same day. Indeed, Buonanno et al [29] showed that individuals from the same household have different exposure profiles. Participation was possible in either French or Dutch, the 2 official languages of the region.

### Sample Population

Because of the exploratory purpose of this study, the sample size estimated for the data collection was first based on feasibility in terms of the number of devices available, the timing, and the size of the field team. This led to a target of 600 participants. At the end of the data collection, we had enrolled 490 participants.

### Data Collection

Data were collected with 3 main instruments: a questionnaire, a set of 3 pollution exposure measurement devices, and a microenvironments diary.

#### Questionnaire

Participants completed a self-administered questionnaire (Multimedia Appendices 4 and 5), available in French or Dutch, and on paper or online (Limesurvey). This questionnaire contained standardized questions and questions based on a literature review. It focused on socioeconomic aspects, lifestyle, perception of health, and perception and knowledge of environmental exposure. The standardized questions were for:

- Perceived health: Patient Health Questionnaire (PHQ)-9 [41], PHQ-15 [42], Generalized Anxiety Disorder (GAD)-7 [43]
- Physical activity: International Physical Activity Questionnaire (IPAQ)-7 [44]
- Dietary habits: Inspired by the Food Frequency Questionnaire used in the Belgian Food Consumption Survey 2014 [45]. Several items were added, based on a literature review, to evaluate the exposure to endocrine-disrupting chemicals through food consumption.
- Modern health worries (MHWs): Inspired by the MHW scale [46]. Several items were added to evaluate the potential worries induced by the COVID-19 sanitary crisis.

We asked the participants to complete the questionnaire a few days before wearing the devices to reduce the priming effect. However, as we would not receive their signed consent until later, on the day they started wearing the devices, they were asked to wait before returning the questionnaire. On the day of the research team’s visit, the participants first signed the information and consent form and then received their pseudonym that they added to the questionnaire before submitting it.

#### Portable Devices

Participants wore 3 different measuring devices (Table 1) at the same time for 24 hours, except when sleeping or showering. The measurements started in the late afternoon with the visit of the field team and ended the next day at the same time plus 15 minutes (24 hours and 15 minutes later). For example, a participant would receive the devices on Monday at 5:00 p.m. and wear them until bedtime. They would leave the devices somewhere in the bedroom in order not to wear them during sleep (for comfort) and put them back on the next morning. The measurements would not be stopped until the field team visited on Tuesday at 5:15 p.m.

**Table 1:** Main characteristics of the portable devices used in the ExpoHealth-1 study data collection.

Characteristic	EMDEX II	EMDEX Lite	Airbeam 2	Smartphone
Environmental pollutant measured	ELF-MF^a^ (frequency range broadband: 40-800 Hz) flux density	ELF-MF (frequency range broadband: 40-800 Hz) flux density	PM^b^_1_, PM_2.5_, and PM_10_ concentration	Noise levels intensity
Range	0.01-300 µT	0.01-70 µT	0-300 µg/m^3^	Pink noise
Unit	µT	µT	µg/m^3^	dB
Frequency of data collection (seconds)	3	4	1	1
Duration of measurement	24 hours except sleeping time	24 hours except sleeping time	24 hours	24 hours
Duration of data upload (minutes)	5-10	5-10	5-10	5-10
Dimensions (cm)	16.8 (height) × 6.6 (width) × 3.8 (depth)	11.9 (height) × 6 (width) × 2.5 (depth)	13.2 (height) × 9.8 (width) × 2.8 (depth)	15.6 (height) × 7.3 (width) × 0.8 (depth)
Weight with battery (g)	367	196	176	154
CE^c^ pouch (g; for EMDEX Lite only)	N/A^d^	54	N/A	N/A
Shoulder pouch (g; for EMDEX + smartphone)^e^	57	57	N/A	57
Way of wearing	Carried in the shoulder pouch	Carried in the CE pouch, which is in the shoulder pouch	Fixed around the waist	Carried in the shoulder pouch
Duration of wearing	All the time (24 hours) except when in a steamed environment and except during the night (somewhere in the bedroom)	All the time (24 hours) except when in a steamed environment and except during the night (somewhere in the bedroom)	All the time (24 hours) except when in a steamed environment and except during the night (somewhere in the bedroom)	All the time (24 hours) except when in a steamed environment and except during the night (somewhere in the bedroom)
Battery	9 V	9 V	2000 mAh 3.7 V	3020 mAh
Autonomy	Up to 7 days	6 days	10 hours	26-30 hours
Cost by unit (US $)	2950	1525	250	100

^a^ELF-MF: extremely low-frequency magnetic field.

^b^PM: particulate matter.

^c^CE: European Conformity.

^d^N/A: not applicable.

^e^Each participant wore only 1 shoulder pouch (with 1 EMDEX and 1 smartphone inside).

#### Airbeam 2 for Particulate Matter

We used Airbeam 2 to collect daily data on exposure to PM_1_, PM_2.5_, and PM_10_ (particle diameter<1, <2.5, and <10 µm, respectively). Airbeam 2 is an optical particle sensor (Plantower PMS7003) that uses light scattering to measure the ambient concentrations of PM (maximum diameter 1, 2.5, and 10 µm). The measured data are then sent to a smartphone via the Aircasting Android app through a Bluetooth connection.

Airbeam 2 quality tests were performed independently of this study by the Air Quality Sensor Performance Evaluation Centre (AQ-SPEC) [47]. Their measurements were compared under laboratory conditions [48] with a federal (US) equivalent method device (FEM GRIMM) and under field conditions [49] with several instruments (MetOne BAM, GRIMM, and Teledyne). No correlation with PM_10_ data was found in either situation. PM_1_ and PM_2.5_ comparisons with reference instruments under laboratory conditions showed a high correlation (R^2^>0.99), but Airbeam 2 underestimated the PM concentrations (accuracy between 62% and 83% for PM_1_ and between 51% and 78% for PM_2.5_) [48]. Under field conditions, the correlations of the measurements with the reference instruments were moderate to very strong for PM_2.5_ (0.67<R^2^<0.86) and strong for PM_1_ (R^2^=~0.75). In general, the accuracy was considered moderate in the range between 0 and 300 µg/m^3^, with frequent underestimation of the concentrations. Stronger correlations were observed for 24-hour averages (R^2^=~0.94 for PM_1_ and ~0.91 for PM_2.5_ when compared to GRIMM) [49].

The sensors showed high precision in several combinations of relative humidity and high or low temperature. In addition, there was little variation between the different Airbeam 2 devices used in the tests. This suggests that we can be confident in the precision of the set of 13 different Airbeam 2 sensors used in the data collection.

In conclusion, we will not be mobilizing the data collected for PM_10_ concentrations as we have low confidence in the devices for this. A correction equation will be developed and applied before further analysis of measured data of PM2.5 concentrations. This will tackle the systematic underestimates observed for Airbeam 2 sensors.

**Figure 2:**
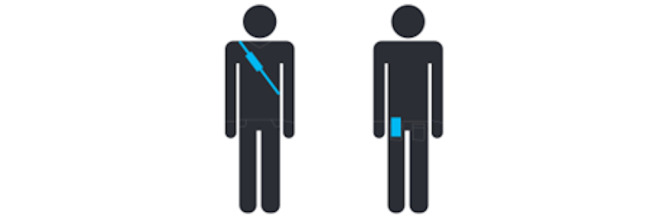
How to wear the portable devices for the ExpoHealth-1 study: EMDEX II or EMDEX Lite + smartphone in a shoulder pouch (either on the front or on the back) and Airbeam 2 at the waist level with access to ambient air.

Airbeam 2 is worn on the belt, with access to ambient air (Figure 2 and Multimedia Appendix 6). It has an autonomy of 8-9 hours. Participants wore it from the end of the afternoon until the evening, recharged it during the night (sleeping hours), and put it back on during the daytime (sometimes with a small charge of 30 minutes during the day and while still wearing it). Occasionally, the device would stop working and display a flashing red light. Participants were asked to call the team if this happened, in which case they reset the device together over the phone at any time of the day or night to minimize the loss of data. The devices measured exposure continuously over 24 hours, even while Airbeam 2 was charging.

#### EMDEX II and EMDEX Lite for ELF-MFs

EMDEX II and EMDEX Lite measure the levels of ELF-MFs. They have been used for many years in ELF-MF-monitoring studies. In 2004, Brazzale et al [50] reviewed their quality. They concluded that both instruments provide “reliable measurements of the reference ELF-MFs.” Comparing the 2 devices, they found that the EMDEX II meters showed a consistent underestimation of 0.01 µT, while the EMDEX Lite measurements showed no fixed error, but the authors observed a relative error influenced by the orientation of the instrument. A relative error of 3.5% (95% CI 3.0%-4.0%) was reported for both instruments, which is slightly higher than the 1%-2% indicated by the manufacturer. In 2002, McDevitt et al [51] used EMDEX Lite to assess the measurement quality of the then brand-new MultiWave System III. They reported similar results in terms of the time-weighted average (TWA) but noticed that EMDEX Lite tends to underestimate the maximum exposure measurements.

A calibration control report on EMDEX Lite used in the study showed important relative errors for exposure levels under 0.1 µT [52]. Measurement levels were compared to those of the reference instrument SMP2 Wavecontrol (probes WP400 and WP400-3).

EMDEX II was compared to a Maschek ESM-100 probe by Geuzaine et al [53]. They found similar results: EMDEX II values are too imprecise under 0.1 µT, and values below this threshold should be considered noise.

Although EMDEX II was developed in 1991, it is still widely used in environmental studies, often for specific microenvironments, such as children’s bedrooms and schools [54], residential rooms categorized according to their vicinity to transformer rooms [55], classrooms during digital learning classes [56], and daily microenvironments of adolescents [57], or to assess ELF-MF on land around high-voltage power lines [58] or generated by portable fans [59].

We used both EMDEX II and EMDEX Lite to assess the exposure of individuals to ELF-MFs in each microenvironment they visited during data collection. EMDEX II was calibrated before the start of data collection. Midway through the field experiment, EMDEX II had to be replaced by a set of EMDEX Lite, all of which were then checked in all 3 directions with the testing suitcase (see pictures in Multimedia Appendix 7) and showed values within the acceptable ranges in all 3 directions. As EMDEX Lite is known to be sensitive to radiofrequency (RF) fields that can be generated by a mobile phone within a 30 cm radius, it was carried in a special European Conformity (CE) anti-RF shielding pouch. As we used a smartphone to record noise levels, we needed this shielding pouch to avoid interference. We noted that the shielding pouch did not affect the measurements of 50/60 Hz MFs.

Each participant was asked to wear the device in the same way (ie, over the shoulder), as shown in Figure 2 and Multimedia Appendix 6, and to not leave it lying around (eg, on a desk or couch). This ensured the quality of the measurements.

As participants did not wear the device during their sleeping hours, the data from these periods will not be used.

The fixed error of –0.01 µT in EMDEX II measurements will be corrected when cleaning the data. Measurements below 0.1 µT will be considered background noise.

#### Smartphones for Noise Levels

Huawei Y6 2019 smartphones (model MRD-LXI) and the Aircasting app were used to (1) measure sound levels in decibels every second, (2) save the data collected with Airbeam 2 and sent to the smartphones via a Bluetooth connection, and (3) record the GPS data of each participant’s movements.

The Aircasting app uses a weighting filter (A), which is most commonly used for environmental noise measurements and is close to the range of human hearing. Therefore, we uploaded the data in dB(A).

Although smartphones are not designed for research purposes, they can collect a large amount of data at an individual level and at low cost [25].

Quality control was performed on the smartphones used for data collection by the noise department of Brussels’ environmental agency (Bruxelles Environnement/Brussel Leefmilieu). The sound levels collected by the Huawei Y6 2019 smartphones via the Aircasting app were compared to the data collected by a reference sonometer of category 1: the Cube from the 01dB brand. A pink sound was used for comparison because it gathers a wide spectrum of sound frequencies and is therefore similar to environmental noise. The tests were conducted under similar conditions to those of the study: in the shoulder pouch and against the EMDEX device. This quality test concluded that the smartphones tended to underestimate the noise levels by 3 dB in comparison to the reference instrument. Therefore, the data collected will be corrected with +3 dB for each take. Furthermore, there was a maximum difference of 1 dB between the different smartphones.

The mobile phone was placed in the shoulder pouch next to the EMDEX device. The participants were asked to always keep a maximum of 1 m and a minimum of 10 cm between Airbeam 2 and the pouch (smartphone + EMDEX). The maximum distance of 1 m ensures a strong Bluetooth connection between Airbeam 2 and the mobile phone. The minimum distance of 10 cm avoids possible interference of the Bluetooth connection with the ELF-MF measurements by EMDEX II.

#### Diary of Microenvironments

Participants filled in a paper microenvironment diary (Multimedia Appendix 8) with a code corresponding to the type of environment they were in (eg, kitchen, car, park, restaurant, workplace) for each 15-minute time slot. Each diary was collected and encoded in a digital database, and 10% of the diaries were randomly checked for possible coding errors.

The microenvironments listed were both indoor (kitchen, living room, workplace, car, public transport) and outdoor (green space, walking or cycling in the street).

#### Organization of the Data Collection

Participants were invited to book an initial appointment between Monday and Thursday afternoons and a second appointment 24 hours later, between Tuesday and Friday afternoons. They were asked to choose a day that was the most representative of their weekly life. Once they had booked an appointment, they received the questionnaire online or on paper (Multimedia Appendices 4 and 5), depending on their preference, and instructions on how to use the measuring equipment (Multimedia Appendix 9).

The research team could meet up to 6 new participants each day and collect up to 6 sets of devices from participants who had started the day before.

Each initial appointment began with a discussion to ensure that all important information was clear to the participant. This was followed by them cosigning the information and consent form. Each participant was given a pseudonym to associate with their filled questionnaire and was asked to submit it immediately. At this point, the devices were started and attached to the participant with explanations to ensure that they were worn correctly: a distance had to be maintained to avoid interference, and the device measuring air pollutants had to always have access to ambient air.

Since part of the data collection took place during the COVID-19 pandemic, hygiene and distance rules were strictly adhered to: Every encounter took place at the doorstep, outside the participants’ homes, and the team always wore a mask and disinfected their hands and the equipment before and after every encounter.

To minimize the environmental impact of data collection, reusable batteries were used throughout the data collection and no more than 20 different 9 V batteries were used in total. Most of the journeys between participants’ homes were made by bicycle or on foot.

### Data Analysis

Data analysis will be mainly descriptive using R software version 4.2.3 (R Foundation for Statistical Computing). The final database will gather the following data for each participant: (1) exposure levels for noise, PM_2.5_, and ELF-MFs in means per minute over 24 hours, (2) the microenvironment in which each exposure was measured, and (3) answers to the questionnaire, which combines personal information, perceived health, perceived exposure, and several life and home habits.

#### Description of Exposures by Participants and by Microenvironment

We will first report the central trend of exposures for each pollutant along the 24-hour measurements. We will then report trends by microenvironment to identify potential differences in exposure between microenvironments and by aggregated indoor and outdoor environments. We will present means (SDs) for normal distributions and medians (IQRs) for nonnormal distributions. We will estimate the contribution of each microenvironment in the levels of exposure with a mass-time-ratio calculation [60] and integrate the time spent in each microenvironment with an integrated exposure calculation [61].

#### Assessment of the Role of Each Microenvironment in the Exposure Levels Measured

Because the data on exposures may be partly dependent (as some data were taken from the same microenvironments of the same participants), we will perform a multilevel analysis (linear mixed model) to evaluate the role of microenvironments in the levels of exposure measured for each pollutant of interest. Control variables, such as gender, age, and period of participation, will be included in the analysis: Y=levels of exposure in means per minute and Xi=microenvironment, gender, age, participation during or after the COVID-19 period.

#### Assessment of the Role of Measured Exposure in Perceived Exposure

Finally, we will test the potential relationship between measured and perceived exposures for each pollutant of interest with linear models, including several control variables, such as age, gender, participation during or after the COVID-19 period, and MHWs. More advanced methods, such as structural equation modeling, will be used to test for mediation between different kinds of exposures with perceived exposures through MHWs and potential interactions.

### Ethical Considerations

The study protocol was approved by the Erasme-ULB Hospital Ethics Committee (approval number P2018/454; approval date November 6, 2018). Consent was obtained from all participants, who signed the information and consent forms. Participants had the option to opt out at any time during data collection. Each participant’s data were associated with a pseudonym. A separate table linking each participant’s personal information (eg, address and phone number) to their pseudonym was created on a fixed computer and made accessible to the researchers’ team only. No compensation was offered to participants.

## Results

Data collection was carried out from October 2020 to August 2022, with several interruptions due to the COVID-19 pandemic. In total, 490 people participated in the data collection. The databases have been gathered and cleaned. The next step is the data analysis, as described before. See Table 2 for the composition of the sample (n=459, 93.7%, of 490 participants who sent back the questionnaire).

**Table 2:** Description of the sample (N=459).

Variables			Participants, n (%)
**Gender**			
	Female	297 (64.7)	
	Male	162 (35.3)	
**Age group (years)**			
	18-29	24 (5.2)	
	30-39	143 (31.2)	
	40-49	151 (32.9)	
	≥50	141 (30.7)	
**Equivalized net household income**			
	<1500 euros (<US $1741)	145 (31.6)	
	1501-2500 euros (US $1742-$2901)	248 (54.0)	
	≥2501 euros (≥US $2903)	66 (14.4)	
**Education**			
	Secondary school (lower and upper levels)	36 (7.8)	
	University (bachelor’s degree, master’s degree, PhD)	423 (92.2)	

## Discussion

### Summary

The objective of this study is to assess personal and simultaneous exposures to multiple pollutants (noise, PM_2.5_, and ELF-MFs) over a normal weekday, with detailed exposure levels for the main microenvironments encountered (home, means of transportation, workplace).

In Brussels, daily averages of exposure to noise and air pollutants are freely accessible from the Brussels environmental agency (Bruxelles Environnement/Brussel Leefmilieu) [62] and the interregional environmental unit IRCELINE [63] websites, respectively. These exposures are measured by several fixed-site monitoring stations distributed in the region and then modeled to estimate levels of exposure in the entire region.

To the best of our knowledge, this study is the first assessment of Brussels’ inhabitants’ personal exposure to multiple pollutants. The results will provide estimates of personal as well as microenvironmental trends of exposure to noise, PM_2.5_, and ELF-MFs for inhabitants of the areas studied. This is a first step toward a detailed picture of citizens’ profiles of multiple-pollutant exposures and toward identifying the main microenvironments affected by these 3 pollutants in Brussels.

The study results will be transmitted to the relevant public institutions: the 3 municipalities in which the data collection took place, the Brussels environmental agency (Bruxelles Environnement/Brussel Leefmilieu), and the federal agency for public health Sciensano.

Every participant in the study has already received their personal results in the shape of averages per 24 hours of measurements. Many of them have shown a strong interest in knowing the levels to which they are exposed in their daily lives. This shows the importance of citizen science.

### Strengths

The main strength of this study is that the exposure levels for several pollutants were collected at an individual level, which provides a much higher level of detail than extrapolated data. This, together with the microenvironment diary, the time of the day, and the profile of the participating population, will allow us to identify factors that can influence the levels of exposure, and therefore to highlight concrete targets to reduce exposures. As the measurements were taken during typical days of the week, they reflect the real-life exposures of the participants. Finally, the study focuses on 3 types of daily exposures measured simultaneously. This responds to the needs underlined by the European Environmental Agency to focus on multiple-pollutant exposures [2].

### Limitations

The main limitation of the study is the lack of representativeness of the participants due to the nonprobabilistic sampling method. As participation in the data collection was voluntary and without any form of reward, the participating individuals appeared to show similar socioeconomic levels, especially in the level of education received. This makes it impossible to test for differences in socioeconomic status. Moreover, no causal relationship will emerge from this study because of its cross-sectional design.

Second, data collection took place only once, during a 24-hour period, for each participant. This will prevent us from drawing any conclusion about long-term exposure.

Moreover, to gather these many data on multiple-pollutant exposures at the individual level, the best option is often low-cost measuring devices. Although the noise and ELF-MF levels measured will be corrected with a simple correction factor, the levels of PM_2.5_ measured with Airbeam 2 will need a corrective equation. Indeed, these devices show systematic underestimations of PM_2.5_ concentrations. A corrective equation adapted to the equipment used in the study is being developed from linear models after colocations of the Airbeam 2 devices and a reference instrument (DustTrak 8534 TSI).

In our data collection for exposure to PM, we did not include a real-time measurement of physical activity, as has been done by other authors [25,64]. This would have allowed us to assess the inhalation rate and therefore the true exposure to air pollutants.

During this data collection, a new model of Airbeam was launched. Airbeam 3 seems to be significantly more efficient and accurate than Airbeam 2. This newer version would have potentially improved our data quality.

Finally, this study focuses on measured exposures and perceived exposures. A cohort design would be more relevant to explore potential links between multiple-pollutant exposures and health impacts.

### Conclusion

This paper aims to describe, with a high level of detail, the methodology and protocol for the assessment of individual exposure to noise, PM, and ELF-MFs for 24 hours on a normal weekday, applied to 490 inhabitants of the Brussels Capital Region. We hope that this detailed description of the protocol will make it easily reproducible, foster further improvements to multiple-pollutant exposure methodologies, and permit a wider use of the database produced. The database created will be shared after the publication of the first results. Characterizing the multiple-pollutant exposures faced by individuals in different microenvironments in Brussels could lead to improved understanding of the sources of exposure and contribute to creating better policies to face multiple-pollutant exposures.

## Appendix

Multimedia Appendix 1 Table of indicators for populations in the areas of interest: socioeconomic indicators and exposure to pollutants for 2019. PM: particulate matter.

Multimedia Appendix 2 Flyer of invitation in French and Dutch.

Multimedia Appendix 3 Posts on social media.

Multimedia Appendix 4 Questionnaire for the ExpoHealth-1 study in French.

Multimedia Appendix 5 English version of the questionnaire for the ExpoHealth-1 study.

Multimedia Appendix 6 Pictures showing how to wear the devices.

Multimedia Appendix 7 Testing suitcase for the measurement levels of ELF-MFs using EMDEX II and EMDEX Lite meters. ELF-MF: extremely low-frequency magnetic field.

Multimedia Appendix 8 Diary of microenvironments.

Multimedia Appendix 9 Procedure for participants (English).

## Abbreviations

BBEMG: Belgian Bioelectromagnetics Group
CE: European Conformity
COPD: chronic obstructive pulmonary disease
ELF-MF: extremely low-frequency magnetic field
EU: European Union
MF: magnetic field
MHW: modern health worry
PHQ: Patient Health Questionnaire
PM: particulate matter
WHO: World Health Organization
