# Supplementary figures and images for: Assessment of Individual Exposure to Multiple Pollutants (Noise, Particulate Matter, and Extremely Low-Frequency Magnetic Fields) Related to Daily Life Microenvironments in the Brussels Capital Region: Protocol for a Cross-Sectional Study
Source: JMIR Res Protoc. 2025 Jul 3;14:e69407. doi: 10.2196/69407 (PMC12271967; doi:10.2196/69407)

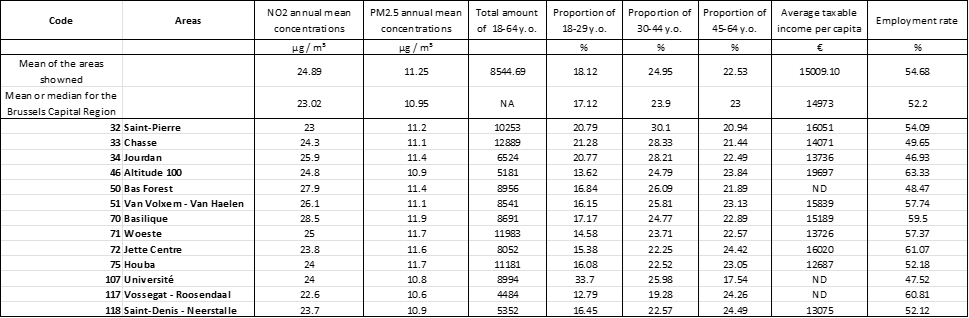

Supplement: Multimedia Appendix 1 [file resprot_v14i1e69407_app1.png]

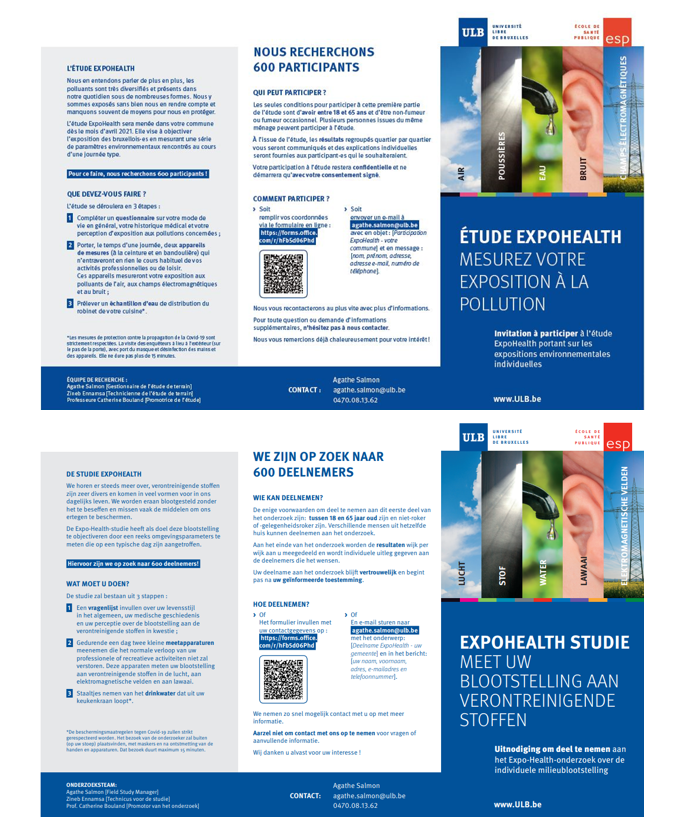

Supplement: Multimedia Appendix 2 [file resprot_v14i1e69407_app2.png]

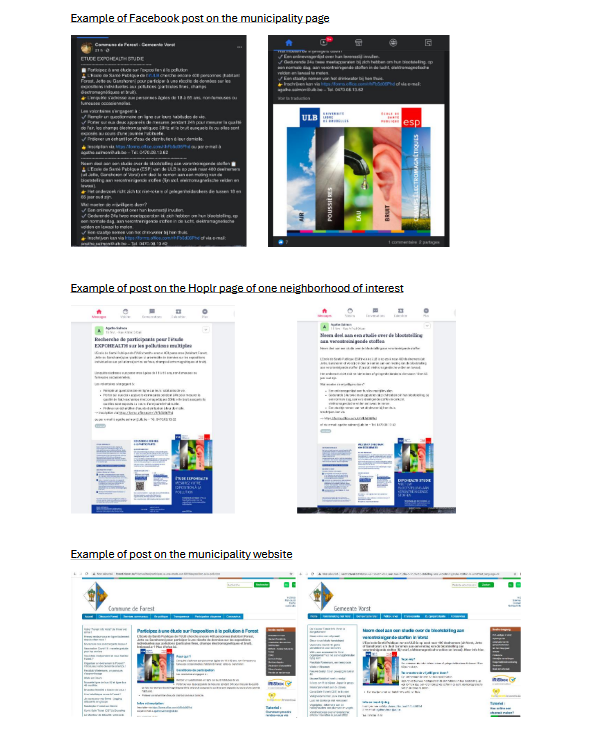

Supplement: Multimedia Appendix 3 [file resprot_v14i1e69407_app3.png]

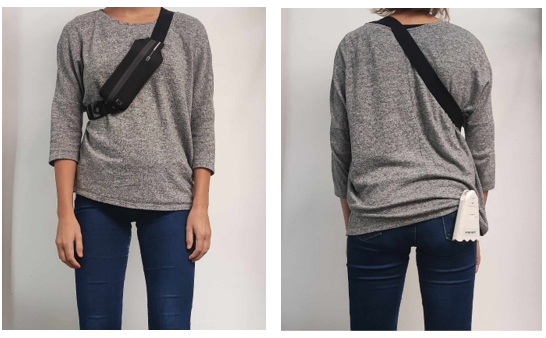

Supplement: Multimedia Appendix 6 [file resprot_v14i1e69407_app6.png]

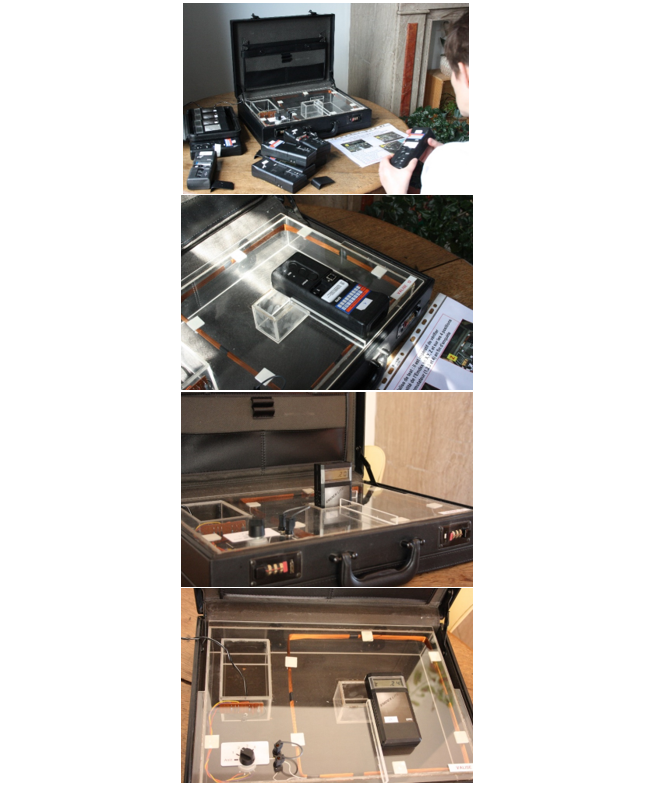

Supplement: Multimedia Appendix 7 [file resprot_v14i1e69407_app7.png]

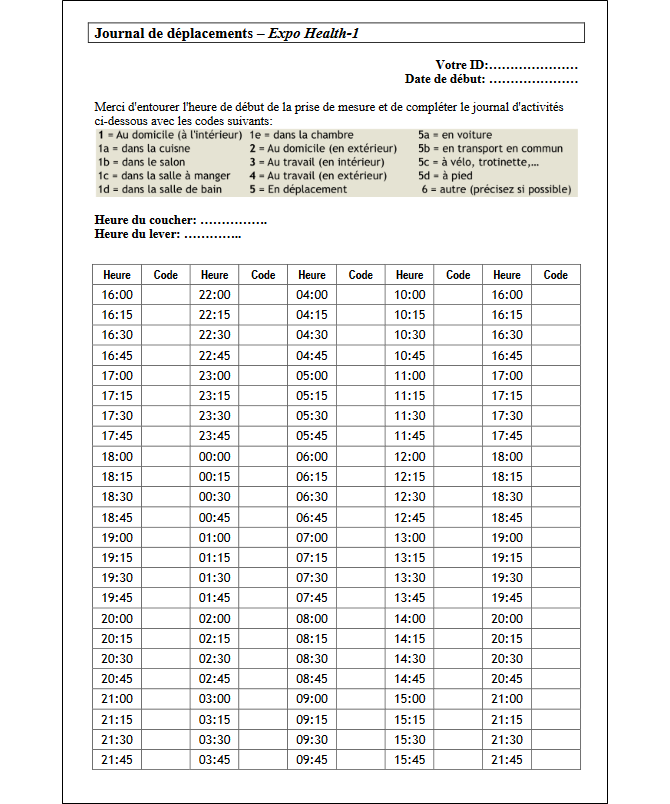

Supplement: Multimedia Appendix 8 [file resprot_v14i1e69407_app8.png]
