# Supplementary material for: Assessment of Individual Exposure to Multiple Pollutants (Noise, Particulate Matter, and Extremely Low-Frequency Magnetic Fields) Related to Daily Life Microenvironments in the Brussels Capital Region: Protocol for a Cross-Sectional Study
Source: JMIR Res Protoc. 2025 Jul 3;14:e69407. doi: 10.2196/69407 (PMC12271967; doi:10.2196/69407)
Supplement: Multimedia Appendix 4 [file resprot_v14i1e69407_app4.pdf]

Etude ExpoHealth-1

Evaluation de l'exposition aux champs magnétiques 50 Hz, aux polluants dans l'air, aux perturbateurs endocriniens et au bruit à Bruxelles, et relation avec la qualité de vie, la santé et la perception des risques

Coordonnées de l'équipe coordinatrice de la recherche :

ULB - Campus Erasme
Centre de Recherche en santé environnementale et santé au travail
Prof. Catherine Bouland - catherine.bouland@ulb.ac.be - 02 555 40 27
Route de Lennik, 808 | 1070 Bruxelles

Enquêteuses :

Agathe Salmon – agathe.salmon@ulb.be

Zineb Ennamsa – zineb.ennamsa@ulb.ac.be

Maryse Ledent – maryse.ledent@sciensano.be

Morgane Eggen – morgane.eggen@ulb.ac.be

Fanny Brunin – fanny.brunin@ulb.ac.be

Questionnaire à rendre à l'enquêteuse après les 24 heures de mesures ou à envoyer à :

Agathe Salmon (A.2.134)
Bâtiment A (ESP, ULB) – CP 593
Route de Lennik, 808
1070 Anderlecht
agathe.salmon@ulb.ac.be

Date du jour : (jour, mois, année) ……/……/ …….

Code (à compléter par les enquêteurs) ………………

Vous remarquerez que ce questionnaire est rédigé au masculin, ce choix a été posé dans le but d'alléger votre lecture. L'ensemble des questions s'adressent évidemment autant aux femmes qu'aux hommes.

L'ULB se conforme au Règlement général sur la protection des données (RGPD) et attache une grande importance à la protection de vos données à caractère personnel. Cela signifie que vos coordonnées préalablement communiquées ainsi que les informations à caractère personnel que vous nous communiquerez dans ce questionnaire seront conservées de manière sécurisée et consultables uniquement par l'équipe de recherche du projet et pour la réalisation de celui-ci. Elles ne seront en aucun cas communiquées à des tiers. En outre, une procédure a été mise en place pour garantir que les données que vous pré-remplirez en ligne resteront anonymes et ne seront pas consultées par l'équipe de recherche avant l'obtention de votre consentement final à participer à la recherche. Si vous décidez de ne pas participer, vos données personnelles seront supprimées.

Toutes les questions relatives à cette recherche peuvent être adressées à *agathe.salmon@ulb.be*, doctorante en charge du projet. Toutes les questions sur la protection de vos données par l'ULB peuvent être envoyées au Délégué à la protection des données : *rgpd@ulb.be*.

Veuillez lire attentivement les questions suivantes et y répondre seul et aussi complètement et précisément que possible. Le questionnaire étant long (+-1h), **n'hésitez pas à faire des pauses et à y revenir plus tard si votre attention s'estompe**.

Nous vous remercions chaleureusement pour votre intérêt et votre participation à cette étude.

CONSENTEMENT ECLAIRE

Questionnaire dans le cadre de l'étude Expo Health

Ce questionnaire fait partie de l'Étude ExpoHealth qui vise l'évaluation de l'exposition aux champs magnétiques 50 Hz, aux polluants dans l'air, aux perturbateurs endocriniens et au bruit à Bruxelles, et la relation de ces expositions avec la qualité de vie, la santé et la perception des risques. Ce questionnaire en ligne prend place au sein de l'étude Expo Health. Vous êtes invité à pré-remplir ce questionnaire en amont de notre visite à votre domicile et du lancement des mesures sur 24h de vos expositions. ***Votre participation à ce questionnaire et au reste de l'étude est volontaire et doit rester libre de toute contrainte. Vous pouvez arrêter votre participation à tout moment et sans justification*** *en informant l'investigatrice (agathe.salmon@ulb.be).*

(Cette question est obligatoire)
CE1. Je déclare poser mon accord libre et informé pour participer à ce questionnaire dans le cadre de l'étude Expo Health menée par le *Centre de Recherche en Santé Environnementale et Santé au Travail de l'Ecole de Santé Publique de l'ULB*. J'ai compris que ma participation à ce questionnaire est volontaire et que je peux l'arrêter à tout moment sans justification. J'ai compris que mes réponses à ce questionnaire seront pseudonymisées et protégées. Les informations fournies ne seront utilisées que pour des statistiques générales qui pourront donner lieu à des publications scientifiques; mais mes données individuelles ne seront pas rendues publiques.

Je consens de mon plein gré à participer à ce questionnaire.

- ○ Oui → Passez à la page suivante
- ○ Non → “ Nous vous remercions pour votre intérêt et vous invitons à informer l'investigatrice principale que vous ne désirez plus participer à l'étude Expo Healh en lui envoyant un email à agathe.salmon@ulb.be. “

DONNEES PERSONNELLES

DP1 Vous êtes : un homme / une femme / autre ……….

DP2 Quelle est votre année de naissance ?

DP3 Quelle est votre nationalité ? ……………….

DP4 Vous êtes originaire de (question d'ordre génétique) :

- ○ Europe
- ○ Afrique du Nord
- ○ Asie
- ○ Amérique du Nord
- ○ Amérique centrale ou du Sud
- ○ Afrique subsaharienne
- ○ Moyen-Orient
- ○ Océanie

DP5 Quel est votre niveau de diplôme le plus élevé

- ○ Non diplômé ou titulaire du certificat d'études de base
- ○ Diplômé de l'enseignement secondaire inférieur
- ○ Diplômé de l'enseignement secondaire supérieur
- ○ Etudiant ou diplômé de l'enseignement supérieur de type court (bachelier)
- ○ Etudiant ou diplômé de l'enseignement supérieur de type long (master)
- ○ Doctorant ou diplômé du 3ème degré de l'enseignement (doctorat)

DP6 Comment se compose votre ménage (personnes habitant au domicile) ?

Nombre d'adultes : ……

Nombre d'enfants : ……

DP7 Dans quelle tranche se situe le revenu net imposable mensuel <u>de votre ménage</u> ?

- ○ En-dessous ou égal à 1500 eur
- ○ Entre 1500eur et 3500 eur
- ○ Entre 3501eur et 5000 eur
- ○ Plus de 5000eur

DP8 Si vous vivez en cohabitation/colocation, combien des personnes habitant sous le même toit dépendent des revenus stipulés ci-dessus ? ………

INFORMATIONS PROFESSIONNELLES

IP1 Statut professionnel

Exercez-vous actuellement une activité professionnelle ? Oui / Non

Si oui : Dans quelle proportion de temps travaillez-vous? ………%

Avez-vous un horaire particulier (type travail posté -horaires rotatifs- ou de nuit) ? Oui / Non. Si oui, quel(s) horaire(s) habituel(s) ? ……………………………………………..

Si non : Quel est votre statut actuel ?

- ○ Étudiant
- ○ Homme ou femme au foyer
- ○ Au chômage
- ○ Incapacité/maladie/congé de maternité, d'allaitement ou parental
- ○ En pause carrière
- ○ (Pré-)pensionné
- ○ Autre:……………

IP3 Dans quel(s) secteur(s) d'activités avez-vous travaillé durant les 10 dernières années ? Vous trouverez les exemples des principaux secteurs d'activité ci-dessous. Merci de préciser votre fonction pour chaque emploi.

EXEMPLES de <u>secteurs d'activité</u>:

- *Hébergement et restauration*
- *Information et communication*
- *Activités scientifiques et techniques*
- *Activités de services administratifs*
- *Enseignement*
- *Santé humaine*
- *Arts, spectacle et activités récréatives*
- *Sport*
- *Vente*
- *Service informatique*
- *Transport et entreposage*
- *Construction*
- *Agriculture/sylviculture/pêche*
- *Industrie manufacturière*
- *Production et distribution d'électricité ou de gaz*
- *Commerce ou réparation de véhicules auto-moto*
- *Sans emploi ou étudiant*
- *Autres: ………………*

Années	Secteur d'activité	Fonction
2020		
2019		
2018		
2017		
2016		
2015		
2014		
2013		
2012		
2011		
2010		

LIEU DE RÉSIDENCE

LR1 En quelle année avez-vous emménagé à cette adresse ? | | | | |

LR2 De quel type de domicile s'agit-il ?

- ○ Maison unifamiliale quatre façades
- ○ Maison unifamiliale trois façades
- ○ Maison unifamiliale mitoyenne
- ○ Immeuble à appartements
  - → A quel étage habitez-vous ?....................
- ○ Autre : ……………….

LR3 Combien y a-t-il de pièces de vie dans le logement *(ne comptez que les chambres et espaces de vie type salon et salle à manger. Ne comptez pas la cuisine, salle de bain, WC, couloirs, ni débarras)* : ……… pièces de vie.

SENSIBILITÉ À L'ENVIRONNEMENT

SE1 Diriez-vous que vous êtes <u>sensible</u> aux paramètres environnementaux suivants ?

	Pas du tout	Légèrement sensible	Moyennement sensible	Très sensible	Extrêmement sensible		Je ne sais pas
Bruit du voisinage	○	○	○	○	○		○
Bruit du trafic	○	○	○	○	○		○
Bruit faible et continu (ex: néons, système d'aération,..)	○	○	○	○	○		○
Champs électromagnétiques générés par les appareils électriques	○	○	○	○	○		○
Champs électromagnétiques générés par le réseau électrique	○	○	○	○	○		○
Champs électromagnétiques des antennes GSM	○	○	○	○	○		○
Gaz d'échappements	○	○	○	○	○		○
Mauvaise qualité de l'air en général	○	○	○	○	○		○
Pesticides dans les aliments	○	○	○	○	○		○
Autres: …………………	○	○	○	○	○		○
…………………	○	○	○	○	○		○

PERCEPTION DE LA SANTÉ ET SYMPTÔMES

PS1 Dans l'ensemble, comment est votre état de santé :

- ☐ Très bon
- ☐ Bon
- ☐ Moyen
- ☐ Mauvais
- ☐ Très mauvais

PS2 Au cours des quatre dernières semaines, à quel point les problèmes suivants vous ont-ils perturbé ? Symptômes physiques

**Symptômes physiques**	Pas du tout perturbé	Un peu perturbé	Beaucoup perturbé
Douleurs au ventre	○	○	○
Maux de dos	○	○	○
Douleurs dans les membres ou les articulations	○	○	○
Douleurs menstruelles ou autres problèmes liés aux règles (pour les femmes seulement)	○	○	○
Douleurs ou problèmes durant les rapports sexuels	○	○	○
Migraines	○	○	○
Douleurs thoraciques	○	○	○
Vertiges	○	○	○
Syncopes, évanouissements	○	○	○
Palpitations	○	○	○
Essoufflement	○	○	○
Constipation, selles molles, diarrhées	○	○	○
Nausée, flatulences ou indigestion	○	○	○
Acouphènes	○	○	○

PS3 Au cours des deux dernières semaines, à quelle fréquence avez-vous souffert des problèmes suivants ? Symptômes de stress

	Jamais	Plusieurs jours	Plus de la moitié du temps	Presque tous les jours
Un sentiment de nervosité, d'anxiété ou de tension	○	○	○	○
Une incapacité à arrêter de s'inquiéter ou à contrôler ses inquiétudes	○	○	○	○
Une inquiétude excessive à propos de certaines choses	○	○	○	○
Des difficultés à se détendre	○	○	○	○
Une agitation telle qu'il est difficile de tenir en place	○	○	○	○
Une tendance à être facilement contrarié ou irritable	○	○	○	○
Un sentiment de peur comme si quelque chose de terrible risquait de se produire	○	○	○	○

PS4 Au cours des deux dernières semaines, à quelle fréquence avez-vous souffert des problèmes suivants ? Symptômes de l'humeur

**Symptômes de l'humeur**	Jamais	Plusieurs jours	Plus de la moitié du temps	Presque tous les jours
Peu d'intérêt ou de plaisir à faire les choses	○	○	○	○
Se sentir triste, déprimé ou désespéré	○	○	○	○
Difficultés à s'endormir ou à rester endormi, ou sommeil excessif	○	○	○	○
Se sentir fatigué ou avoir peu d'énergie	○	○	○	○
Avoir peu d'appétit ou trop manger	○	○	○	○
Avoir une mauvaise image de vous – penser que vous êtes en échec, que vous avez déçu vos attentes ou celles de votre famille.	○	○	○	○
Difficultés à se concentrer sur des choses telles que lire le journal ou regarder la télévision	○	○	○	○
Bouger ou parler si lentement que les autres personnes pourraient le remarquer. Ou au contraire, être si agité ou fébrile que vous bougez beaucoup plus que d'habitude.	○	○	○	○
Penser que vous seriez mieux mort ou songer à vous blesser d'une façon quelconque.	○	○	○	○

PS5 Au cours des deux dernières semaines, à quelle fréquence avez-vous souffert des problèmes suivants ?

	Jamais	Plusieurs jours	Plus de la moitié du temps	Presque tous les jours
Difficultés de concentration	○	○	○	○
Problèmes de mémoire	○	○	○	○
Difficulté à trouver ses mots	○	○	○	○

PERCEPTION DE L'ENVIRONNEMENT

PE1 Évaluez dans quelle mesure vous <u>êtes préoccupé</u> par l'impact des agents suivants sur la santé, que vous y soyez exposé ou non.

	Pas du tout préoccupé	Un peu préoccupé	Moyennement préoccupé	Très préoccupé	Extrêmement préoccupé
Mauvaise ventilation des bâtiments	○	○	○	○	○
Contamination dans l'eau de distribution	○	○	○	○	○
Programmes de vaccination	○	○	○	○	○
Sur-utilisation des antibiotiques	○	○	○	○	○
Substances chimiques toxiques dans les produits ménagers	○	○	○	○	○
Fuites de rayonnement des fours à micro-ondes	○	○	○	○	○
Bactéries dans les systèmes de climatisation	○	○	○	○	○
Bactéries résistantes aux médicaments	○	○	○	○	○
Amalgames pour plombages dentaires	○	○	○	○	○
Radiographies médicales et dentaires	○	○	○	○	○
Pollution de l'air	○	○	○	○	○
Nuisances sonores	○	○	○	○	○
Appauvrissement de la couche d'ozone	○	○	○	○	○
Gaz d'échappement	○	○	○	○	○
Virus SARS-CoV-2 (Covid-19)	○	○	○	○	○
Vaporisateur de pesticides	○	○	○	○	○
Aliments génétiquement modifiés	○	○	○	○	○
Additifs dans les aliments	○	○	○	○	○
Pesticides dans les aliments	○	○	○	○	○
Antibiotiques dans les aliments	○	○	○	○	○
Hormones dans les aliments	○	○	○	○	○
Téléphones portables	○	○	○	○	○
Antennes de radiocommunication ou de téléphonie mobile	○	○	○	○	○
Lignes à haute tension	○	○	○	○	○
Changements climatiques/gaz à effet de serre	○	○	○	○	○
Champs électromagnétiques générés par les appareils électriques	○	○	○	○	○
Champs électromagnétiques générés par le réseau électrique	○	○	○	○	○
Autres : ……………	○	○	○	○	○

PE2 Dans quelle mesure pensez-vous être exposé aux paramètres environnementaux suivants ?

	Pas du tout exposé	Un peu exposé	Moyennement exposé	Très exposé	Extrêmement exposé	Je ne sais pas
Bruits du voisinage						
Bruit du trafic						
Bruit faible et continu (ex: néons, système d'aération,..)						
Champs électromagnétiques générés par les appareils électriques						
Champs électromagnétiques générés par le réseau électrique						
Champs électromagnétiques des antennes GSM						
Gaz d'échappement des voitures						
Pesticides dans les aliments						

PE3 A quelle fréquence avez-vous entendu parler des risques sur la santé des paramètres environnementaux suivants, <u>au cours des 3 derniers mois</u> ? (Tous types de communication confondus : par exemple via des amis, les médias, en rue, etc. <u>sauf via cette étude</u>)

	Jamais (sur les 3 derniers mois)	1 à 2 fois	Plus de 3 fois
Le bruit	○	○	○
Les champs électromagnétiques du réseau électrique	○	○	○
Les champs électromagnétiques des appareils électriques	○	○	○
Les champs électromagnétiques des antennes relais (GSM)	○	○	○
Les perturbateurs endocriniens	○	○	○
La pollution de l'air	○	○	○

PE4 Trouvez-vous qu'il fait bruyant dans votre domicile ? *(Si le bruit est variable, donnez une valeur moyenne)*

Dans mon domicile, je trouve qu'il fait :

1	2	3	4	5

Très silencieux *Très bruyant*

- Si vous trouvez que l'ambiance interne de votre maison/appartement est bruyante, expliquez pourquoi si possible :

  ……………………………………………………………………………………………
  ……………………………………………………………………………………………
  ……………………………………………………………………………………………

PE5 Comment percevez-vous la <u>qualité de l'air intérieur de votre domicile</u> ?
(Si la qualité de l'air intérieur varie, donnez une valeur moyenne)

☐ Je trouve que la qualité de l'air dans mon domicile est :

1	2	3	4	5

Très bonne *Très mauvaise*

- Si vous pensez que la qualité de l'air intérieur n'est pas bonne, expliquez pourquoi si possible :

  ……………………………………………………………………………………………
  ……………………………………………………………………………………………
  ……………………………………………………………………………………………

PE6 Comment percevez-vous la <u>qualité de l'air extérieur aux alentours de votre domicile</u> ? *(Si la qualité de l'air à l'extérieur varie, donnez une valeur moyenne)*

☐ Je trouve que la qualité de l'air aux alentours de mon domicile est :

1	2	3	4	5

Très bonne *Très mauvaise*

- Si vous pensez que la qualité de l'air extérieur n'est pas bonne, expliquez pourquoi si possible :

  ……………………………………………………………………………………………
  ……………………………………………………………………………………………

PE7 Pensez-vous être exposé aux champs électromagnétiques à l'intérieur de votre domicile ? *(Si l'exposition varie, donnez une valeur moyenne)*

☐ Concernant l'exposition aux champs électromagnétiques dans mon domicile, je pense être

1	2	3	4	5

Pas du tout exposé … *Très fortement exposé*

- ➢ Si vous pensez que votre exposition est élevée, expliquez pourquoi si possible :

  ………………………………………………………………………………………………

  ………………………………………………………………………………………………

PE8 Pensez-vous être exposé aux champs électromagnétiques dans votre quartier ?

(Si l'exposition à l'extérieur varie, donnez une valeur moyenne)

☐ Concernant l'exposition aux champs électromagnétiques dans mon quartier, je pense être :

1	2	3	4	5

Pas du tout exposé … *Très fortement exposé*

- ➢ Si vous pensez que votre exposition est élevée, expliquez pourquoi si possible :

  ………………………………………………………………………………………………

  ………………………………………………………………………………………………

ESPACES VERTS : PERCEPTION ET FREQUENTATION

EV1 Votre domicile comprend-il un espace extérieur ?

- ○ Oui un jardin
- ○ Oui un jardin partagé
- ○ Oui une terrasse
- ○ Oui un balcon
- ○ Non

EV2 Y a-t-il des espaces verts dans votre quartier ? [Si vous cochez "pas du tout", passez à la question EV4]

1	2	3	4	5

Pas du tout … *Beaucoup*

EV3 Fréquentez-vous les espaces verts présents dans votre quartier ?

1 2 3 4 5

Jamais *Très souvent (plusieurs fois par semaine)*

EV4 Êtes-vous satisfait du nombre d'espaces verts dans votre quartier ?

1 2 3 4 5

Pas du tout satisfait *Totalement satisfait*

EV5 De manière générale, qualifieriez-vous les rues de votre quartier de « végétalisées » (présence de parterres de fleurs, arbres, buissons, herbes, …) ?

OUI/NON

EV6 Modifiez-vous votre itinéraire lors de déplacements à pied/à vélo pour passer par ce type de rues (fleuries, vertes, aérées) ?

1 2 3 4 5

Jamais *Très souvent*

ANTÉCÉDENTS MÉDICAUX ET GÉNÉTIQUES

MG1 Quel est votre poids ? ………. Kg

MG2 Quelle est votre taille ? ………cm

MG3 Quelle est votre tension habituelle (si vous la connaissez) ? …………/…………

MG4 Souffrez-vous ou avez-vous souffert des maladies suivantes ? Si oui, complétez le tableau

	**Oui /Non**	**Période** Mois et année		**Traitement suivi**	**Précisions sur la pathologie (ex: cancer du sein)**
Cancer	Oui/ Non	De ……/…...	À ……/……		
Hépatite	Oui/ Non	De ……/…...	À ……/……		
Mononucléose	Oui/ Non	De ……/…...	À ……/……		
Herpès	Oui/ Non	De ……/…...	À ……/……		

SIDA	Oui/ Non	De ……/…...	À ……/……		
Méningite	Oui/ Non	De ……/…...	À ……/……		
Infection bactérienne ou virale (récente!)	Oui/ Non	De ……/…...	À ……/……		
Maladie cardiovasculaire	Oui/ Non	De ……/…...	À ……/……		
Diabète	Oui/ Non	De ……/…...	À ……/……		
Allergie respiratoire	Oui/ Non	De ……/…...	À ……/……		
Troubles de la concentration	Oui/ Non	De ……/…...	À ……/……		
Autres: …………………	Oui/ Non	De ……/…...	À ……/……		

MG5 Dressez la liste des radiographies diagnostiques ou thérapeutiques autres que dentaires que vous avez reçues au cours des 5 dernières années. Pensez aux radios et scanners, pas aux échographies ni aux IRM.

Raison(s) des radiographies	Date(s) des radiographies (mm/aaaa)

MG6 Avez-vous eu une radiographie dentaire ? Oui/Non

Si oui, cochez la période :

- o Le mois précédent
- o Dans les 6 mois précédents
- o Dans les 6 à 12 mois
- o Il y a plus d'un an

MG7 Avez-vous connaissance de malformations congénitales ou d'autres troubles génétiques ou maladies héréditaires qui affectent des membres de votre famille biologique ?

Oui / Non

Si oui, quel membre de votre famille ? Cochez la/les personne(s) concernée(s) et précisez le trouble.

Membre de la famille biologique	Nature du trouble
o Mère	………………………………………………
o Père	………………………………………………
o Sœur	………………………………………………
o Frère	………………………………………………
o Enfant	………………………………………………
o Grand-Père	………………………………………………
o Grand-mère	………………………………………………
o Neveu ou nièce	………………………………………………
o Oncle ou tante	………………………………………………

MG8 Concernant les maladies héréditaires :

- o Je suis porteur d'une maladie héréditaire (gène récessif)
  - ➢ Laquelle ? …………………
- o Je souffre d'une maladie héréditaire
  - ➢ Laquelle ? …………………
- o Je ne suis, à ma connaissance, ni porteur, ni atteint d'une maladie héréditaire

MG9 Avez-vous eu des difficultés à concevoir un enfant (période d'au moins 12 mois d'essais non concluants) ?

- o Oui
  - ➢ Si oui, indiquez quand vous avez rencontré cette difficulté :
    De ..…/……… à ……/……… (mois/année)
- o Non
- o Non concerné (c.-à-d., "n'a pas essayé")
  - ➢ *Si vous avez coché "non" ou "non concerné", passez à la question MG13*

MG10 Avez-vous déjà subi un test de fertilité (dosage hormonal, spermogramme, échographie pelvienne, …) ? Oui / Non

MG11 Avez-vous été diagnostiqué(e) comme infertile ? Oui / Non

Si oui, indiquez quand vous avez reçu le diagnostic : …/…… (mois/année)

MG12 Prenez-vous des médicaments hormonaux ?

- Oui :
    - Si oui, précisez :
        - Substituts hormonaux
        - Pilule contraceptive
        - Stérilet ou anneau hormonal
        - Autre : …………….
- Non

MODE DE VIE ET COMPORTEMENTS

Tabagisme

LS1 Avez-vous déjà fumé (plus d'une année) ?

Oui / Non [Si NON, passez à la question LS5]

Si OUI, quel âge aviez-vous quand vous avez commencé à fumer ? ....

LS2 Fumez-vous actuellement ? Oui / Non

Si non, quel âge aviez-vous quand vous avez arrêté de fumer ? ....

LS3 Combien de cigarettes fumiez-vous ou fumez-vous en moyenne par jour ?

- Moins de une
- Entre 1 et 5
- Entre 5 et 20
- Plus de 20

LS4 Fumez-vous autre chose que des cigarettes (ex : pipe à eau, …) ?

- Oui
  - Si oui, que fumez-vous ? ……………….
  - A quelle fréquence fumez-vous ? …………………..
- Non

Drogues

LS5 Consommez-vous ou avez-vous déjà consommé des substances récréatives ?

- Oui / Non
- Si vous en consommez actuellement, de laquelle/desquelles s'agit-il ?
  - Cannabis,
  - Champignons hallucinogènes,
  - Ecstasy/MDMA,
  - Cocaïne,
  - LSD,
  - Amphétamine,
  - GHB,
  - Kétamine,
  - Popper,
  - Autre(s) : ………………)

- Si vous en consommez actuellement, à quelle fréquence le faites-vous ?
  - Plusieurs fois par semaine
  - Entre 1 et 5 fois par mois
  - Entre 5 et 11 fois par an
  - Mois de 5 fois par an

Régime alimentaire (habitudes actuelles)

LS6 Complétez le tableau ci-dessous qui porte sur votre consommation alimentaire étalée sur un an.

<u>Comment remplir le tableau :</u>

Toutes les questions s'intéressent à la moyenne de votre consommation sur 12 mois.

Remplissez donc comme suit :

Ex. 1: Vous *ne mangez jamais mollusques ou crustacés → cochez la case "jamais"*

Ex. 2: Vous mangez de la viande au barbecue, mais seulement en été à raison de 2 fois par semaine environ → Sur un calcul de toute l'année, cela revient à une moyenne de 8 à 10 fois par an, donc moins d'une fois par mois

Ex. 3: Vous buvez du thé tous les 2 jours environ → Cela fait une moyenne de 2-4 fois par semaine

	CONSOMMATION MOYENNE DURANT LES 12 DERNIERS MOIS								
	Jamais	Par mois		par semaine			par jour		
		moins de 1 fois	1-3 fois	1 fois	2-4 fois	5-6 fois	1 fois	2-3 fois	plus de 3 fois
Mollusques ou crustacés	X								
Viande au Barbecue		X							
Thé					X				

Votre Tableau de fréquence alimentaire :

	CONSOMMATION MOYENNE DURANT LES 12 DERNIERS MOIS								
	jamais	par mois		par semaine			par jour		
		moins de 1 fois	1-3 fois	1 fois	2-4 fois	5-6 fois	1 fois	2-3 fois	plus de 3 fois
**VIANDE**									
Viande rouge (ex : bœuf, mouton, agneau, …)									
Viande blanche (ex: veau, volaille, lapin, porc,..)									
Préparations à base de viande transformée (saucisses, hamburgers, nuggets,…)									
Toute viande cuite au barbecue									
**POISSONS et Fruits de mer FRAIS**									
Poisson blanc (ex: cabillaud, lieu, loup de mer, dorade, sole,…)									
Poisson gras (ex: saumon, thon, hareng, turbot,…)									
Mollusques et crustacés (ex: moules, poulpe, palourdes, scampis, …)									
**POISSONS EN CONSERVE**									
Poissons ou fruits de mer en conserve (ex: thon, maquereau, anchois, saumon, sardines, …)									
**BOISSONS**									
café / thé									
softs									
tisane									
bière									
vin									
autres alcools									
**EAU**									
Eau de distribution (non filtrée)									
Eau de distribution filtrée par vos soins (carafe ou autre dispositif filtrant)									
Eau en bouteille en plastique									
**Produits à base de soja**									
Produits à base de soja (ex: tofu, lait de soja, germes de soja, sauce soja,…)									

	CONSOMMATION MOYENNE DURANT LES 12 DERNIERS MOIS								
	jamais	par mois		par semaine			par jour		
		moins de 1 fois	1-3 fois	1 fois	2-4 fois	5-6 fois	1 fois	2-3 fois	plus de 3 fois
**PRODUITS LAITIERS**									
Lait entier									
Yaourt entier									
Fromage à pâte dure									
Fromage à pâte molle									
Fromage blanc									
Beurre									
Autres:……………….									
**PLATS PREPARES**									
Plats préparés (achetés tels quels en magasin)									
Restaurants: Plats livrés ou mangés sur place									
**PRODUITS SUCRANTS**									
Edulcorant artificiel (aspartame,…)									
**CONTENANTS**									
Boissons en canettes									
Produits en conserves métalliques (ex: soupes, raviolis, sauce tomate, choucroute, maïs, etc…)									

**Ce tableau de fréquence alimentaire est inspiré du questionnaire de Sciensano "Enquête de consommation alimentaire belge 2014"*

LS7 Suivez-vous un régime alimentaire spécifique ?

- Oui
    - Si oui, lequel :
        - Hyper protéiné
        - Végétarien
        - Végétalien
        - Faible en lipides
        - Faible en glucides
        - Autre : ……………………
- Non

LS8 Si vous consommez du thé/tisane, sous quelle forme le laissez-vous infuser ? (Plusieurs réponses possibles)

- Dans un contenant en plastique ou en nylon
- Dans un contenant en papier
- Dans un contenant en métal
- En vrac dans l'eau chaude
- Autre : …………………
- Je ne consomme pas de thé, tisane

LS9 Au cours du dernier mois, <u>sur l'entièreté des aliments consommés</u>, quel pourcentage environ était d'origine biologique ? …….%

LS10 Au cours du dernier mois <u>sur l'entièreté des aliments consommés</u>, quel pourcentage environ était emballé dans du plastique ? …….%

LS11 Au cours du dernier mois <u>sur l'entièreté des aliments consommés</u>, quel pourcentage environ était emballé dans des cartons imprimés ? ………%

LS12 Si vous consommez des produits d'origine biologique, où vous les procurez-vous ?

- ❖ Grandes surfaces (type Carrefour, Delhaize, Lidl, Colruyt, Aldi, …)
- ❖ Grandes enseignes labellisées bio (Bioplanet, Carrefour bio, …)
- ❖ Boutique ou épicerie de quartier bio
- ❖ Producteurs locaux, GASAP, paniers bio, etc.
- ❖ Je ne consomme pas bio

LS13 Si vous utilisez une gourde plusieurs fois par semaine,

- ❖ En quelle matière est-elle ?
  - o Elle est en plastique
  - o Elle est en métal/inox
  - o Elle est en verre
  - o Autre (précisez) : ………………
  - o Je n'utilise pas de gourde, ou seulement occasionnellement [passez à la question LS14]

- ❖ Vous y transportez des boissons
  - o Chaudes
  - o Froides
  - o Les deux

LS14 Vos ustensiles de cuisine sont en (plusieurs réponses possibles) :

- o Bois
- o Inox
- o Plastique/silicone
- o Bambou
- o Autre (précisez) : ……………….

LS15 Vous arrive-t-il de réchauffer au micro-onde des aliments dans un récipient en plastique (Tupperware, …) ?

- o Oui, plusieurs fois par semaine.
- o Oui, quelques fois par mois
- o Oui, mais très rarement
- o Non, jamais

Cosmétiques

LS16 Quelle fréquence correspond le mieux à votre utilisation des produits suivants ?

	Tous les jours	Tous les 2 ou 3 jours	1 fois par semaine	1 à 3 fois par mois	Moins d'1fois par mois	Jamais
Savon						
Shampoing						
Après-shampoing						
Produit hydratant pour le corps						
Crème de jour						
Crème de nuit						
Gommages (corps ou visage)						
Mousse à raser/produits dépilatoires						
Après-rasage						
Spray/laque/gel pour cheveux						
Huile cheveux						
Produit démaquillant						
Lotion tonifiante						
Maquillage (mascara, crayon, fond de teint et/ou rouge à lèvres, …)						
Baume à lèvre (hydratant)						
Déodorant						
Parfum						
Vernis à ongle						
Autres : ………………….						

LS17 Vous mettez de la crème solaire :

- o Jamais
- o En période estivale seulement (c.-à-d., en période d'exposition au soleil)
- o Très régulièrement
- o Autre : …….

LS18 Sur l'ensemble de vos produits cosmétiques et de soin, combien sont :

	Faits maisons par vos soins (à base de produits naturels)	Certifié.s "Organique"	Certifié.s "Sans parabène"	Certifié.s "Sans phthalate"	Certifié.s "Avec action antimicrobienne"	Non parfumé.s
**Aucun**	○	○	○	○	○	○
**Moins d'un quart**	○	○	○	○	○	○
**La moitié environ**	○	○	○	○	○	○
**Plus de la moitié**	○	○	○	○	○	○
**Tous**	○	○	○	○	○	○
**Je ne sais pas**	○	○	○	○	○	○

GSM/Smartphones

LS19 Utilisez-vous un GSM/Smartphone ?

Oui/Non

Si oui, Combien d'heures par jour de semaine en moyenne ?

- Moins d'1h
- Entre 1h et 3h
- Entre 3 et 5h
- Plus de 5 h

Combien d'heures par jour de week-end en moyenne ?

- Moins d'1h
- Entre 1h et 3h
- Entre 3 et 5h
- Plus de 5 h

LS20 Comment utilisez-vous votre GSM/Smartphone ?

**Envoi et lecture de SMS**	Oui / Non	Nombre par semaine en moyenne :<br>o 0-10<br>o 11-50<br>o 51-100<br>o Plus de 100
**Conversation téléphonique**	Oui / Non	Nombre d'heures par semaine en moyenne :<br>o Moins d'1h<br>o 1h-5h<br>o 5h-10h<br>o Plus de 10h
**Jeux & réseaux sociaux**	Oui / Non	Nombre d'heures par semaine en moyenne :<br>o Moins d'1h<br>o 1h-5h<br>o 5h-10h<br>o Plus de 10h
**Musique & vidéos**	Oui / Non	Nombre d'heures par semaine en moyenne :<br>o Moins d'1h<br>o 1h-5h<br>o 5h-10h<br>o Plus de 10h
**Agenda & emails**	Oui / Non	Nombre d'heures par semaine en moyenne :<br>o Moins d'1h<br>o 1h-5h<br>o 5h-10h<br>o Plus de 10h
**Autres : ………………**	Oui / Non	Nombre d'heures par semaine en moyenne :<br>o Moins d'1h<br>o 1h-5h<br>o 5h-10h<br>o Plus de 10h

LS20bis Lors des conversations téléphoniques, comment portez-vous votre Gsm/Smartphone ?

	Jamais	Parfois	Souvent	Toujours
GSM à l'oreille	○	○	○	○
Utilisation d'une oreillette Bluetooth	○	○	○	○
Utilisation d'une oreillette filaire	○	○	○	○
Haut-parleur/kit main libre	○	○	○	○

LS21 Quand vous n'utilisez pas votre GSM/Smartphone, où le placez-vous habituellement ? (Plusieurs réponses possibles)

	**Durant vos heures d'éveil**	**Durant vos heures de sommeil**
Dans la poche arrière de votre pantalon/jupe	○	○
Dans la poche avant de votre pantalon/jupe	○	○
Dans votre sac	○	○
Dans votre soutien-gorge	○	○
Dans une sacoche portée à la taille (type banane)	○	○
Il est posé ailleurs dans la pièce où vous vous trouvez	○	○
Il est posé dans une autre pièce que celle où vous vous trouvez	○	○
Près de votre tête (ex ;: sous l'oreiller)	○	○
Autre: ………………………	○	○

LS22 Sur les 7 derniers jours, combien d'heures par jour environ avez-vous utilisé des écouteurs ou un casque pour écouter de la musique/des podcasts, etc… (Utilisation hors appels téléphoniques)

- o Je n'ai pas utilisé d'écouteurs
- o J'ai utilisé des écouteurs environ ……h……min par jour

Activités physiques

Pensez aux activités physiques que vous faites au travail, pour vous déplacer, pendant vos loisirs. Quels types d’efforts fournissez-vous ?

Efforts physiques intenses

AP1 Au cours des 7 derniers jours, combien de fois avez-vous fait des activités physiques vigoureuses comme soulever des charges lourdes, terrasser, pratiquer du fitness (sport en salle) ou rouler rapidement à vélo pendant au moins 20 min ? **………… fois**

AP2 Combien de temps avez-vous consacré à ces efforts physiques intenses, en moyenne les jours où vous avez pratiqué ces activités physiques ?

- o .... minutes par jour où j'ai pratiqué ces activités
- o Je ne sais pas

Efforts physiques modérés

AP3 Au cours des 7 derniers jours, combien de fois avez-vous fait des activités physiques modérées comme porter des charges légères, faire de la bicyclette à un rythme régulier ou du tennis en double pendant au moins 20 min ? Ne pas inclure la marche ! **……. fois**

AP4 Combien de temps avez-vous consacré à ces efforts physiques modérés, en moyenne les jours où vous avez pratiqué ces activités physiques ?

- o .... minutes par jour où j'ai pratiqué ces activités physiques
- o Je ne sais pas

Marche à pied

AP5 Pensez au temps que vous avez passé à marcher au travail et à la maison, à marcher pour vous déplacer d'un endroit à l'autre et à des fins récréatives et/ou sportives. Au cours des 7 derniers jours, combien de fois avez-vous marché au moins pendant 20 min ? **……. fois**

AP6 Combien de temps avez-vous passé à marcher, en moyenne les jours où vous avez marché ?

- o .... minutes par jour
- o Je ne sais pas

AP7 En général, vous marchez à :

- Un rythme intense qui vous fait respirer beaucoup plus fort que la normale
- Un rythme modéré qui vous fait respirer un peu plus fort que la normale
- Un rythme plus lent où il n'y a aucun changement dans votre respiration
- Je ne sais pas répondre

AP8 Qu'est-ce qui décrit le mieux vos activités de loisirs au cours de la dernière année ? (Une seule réponse possible !)

- Entraînement dur et sport de compétition plus d'une fois par semaine
- Jogging et autres sports récréatifs ou jardinage, au moins 4 heures par semaine
- Jogging et autres sports récréatifs ou jardinage, au plus 4 heures par semaine
- Marche, vélo ou autres activités légères au moins 4 heures par semaine
- Marche, vélo ou autres activités légères au plus 4 heures par semaine
- Lecture, regarder la télévision ou autres activités sédentaires
- Je ne sais pas répondre

Déplacements

LS23 Comment vous déplacez-vous généralement pour vous rendre sur les lieux suivants (faites une croix dans la/les cases adéquates ET complétez la dernière colonne) :

	À pied	À vélo	À vélo ou trottinette électrique	En transport en commun (bus, tram, métro, train)	En voiture ou à moto/ mobylette	Mix 1) marche ou vélo + 2) transports en commun	Autre	Précisez la distance et la fréquence à laquelle vous faites ce trajet (si applicable)
Au travail/ à l'école	O	O	O	O	O	O	O	………km<br>………… fois par semaine
A vos loisirs sportifs	O	O	O	O	O	O	O	………km<br>………… fois par semaine
Faire vos courses (lieu habituel)	O	O	O	O	O	O	O	………km<br>………… fois par semaine
Au restaurant, cinéma,<br>sorties, autres activités de détente	O	O	O	O	O	O	O	………km<br>………… fois par semaine
Autre (précisez) : ………………	O	O	O	O	O	O	O	………km<br>………… fois par semaine

LS24 Si vous faites certains trajets à pied, à vélo ou à vélo/trottinette électrique, vous arrive-t-il de modifier votre itinéraire pour éviter :

La pollution de l'air extérieur (ressentie ou avérée) :

- O Parfois
- O Souvent
- O Toujours
- O Jamais

Le bruit lié au trafic :

- O Parfois
- O Souvent
- O Toujours
- O Jamais

LS25 Si vous effectuez certains trajets à pied, à vélo ou à vélo/trottinette électrique : Est-il facile, de manière générale, de vous déplacer avec ces modes de transport dans votre quartier ? (Trottoirs assez larges, zones piétonnes, sécurité du piéton et du cycliste, pistes cyclables, facilité à traverser les grands axes, …)

Oui/Non

Si non, quelles difficultés rencontrez-vous ?

…………………………………………………………………………………………

…………………………………………………………………………………………

…………………………………………………………………………………………

HISTORIQUE DES EXPOSITIONS

HE1 A votre connaissance, avez-vous déjà été exposé à l'un des paramètres suivants dans le cadre de votre travail ou de vos loisirs ? Pensez aux possibles expositions ponctuelles à des taux très élevés et aux expositions chroniques.

	**Oui ou Non ?**	**Si oui, était-ce une exposition ponctuelle ou chronique ?**	**Si oui, à quelle période environ est-ce arrivé ? (mois / année)**		**Si Oui, vous pouvez donner des précisions dans cet espace**
Amiante	Oui/ Non	○ Ponctuelle<br>○ Chronique	De ……../……….	À ………/……..	………………………………………
Radiations ionisantes	Oui/ Non	○ Ponctuelle<br>○ Chronique	De ……../……….	À ………/……..	………………………………………
Poussières (bois, particules métalliques, etc.)	Oui/ Non	○ Ponctuelle<br>○ Chronique	De ……../……….	À ………/……..	………………………………………
Produits de charbon (houille, coke, …)	Oui/ Non	○ Ponctuelle<br>○ Chronique	De ……../……….	À ………/……..	………………………………………
Pesticides/Herbicides	Oui/ Non	○ Ponctuelle<br>○ Chronique	De ……../……….	À ………/……..	………………………………………
Produits pétroliers (essence, mazout)	Oui/ Non	○ Ponctuelle<br>○ Chronique	De ……../……….	À ………/……..	………………………………………
Colorants	Oui/ Non	○ Ponctuelle<br>○ Chronique	De ……../……….	À ………/……..	………………………………………
Solvants	Oui/ Non	○ Ponctuelle<br>○ Chronique	De ……../……….	À ………/……..	………………………………………
Fumées de combustion	Oui/ Non	○ Ponctuelle<br>○ Chronique	De ……../……….	À ………/……..	………………………………………
Autres produits chimiques : ………………	Oui/ Non	○ Ponctuelle<br>○ Chronique	De ……../……….	À ………/……..	………………………………………

ENVIRONNEMENT INTÉRIEUR

EI1 Si votre domicile est équipé d'une cheminée ouverte, l'utilisez-vous ?

- Oui
- Non
- Il n'y a pas de cheminée ouverte dans le domicile

EI2 Quelle est l'énergie principale de cuisson ?

- Electrique
- Gaz
- Charbon ou poêle à bois

EI3 Y a-t-il une hotte aspirante dans la cuisine ?

- Oui et elle est reliée à l'extérieur (appelée " hotte à évacuation")
- Oui, mais elle n'est pas reliée à l'extérieur (appelée "hotte de recyclage")
- Non

EI4 Y a-t-il un chauffage au gaz dans le domicile ?

- Oui et la prise d'air est raccordée à l'extérieur
- Oui et la prise d'air n'est pas raccordée à l'extérieur
- Non

EI5 Combien de cigarettes sont fumées en moyenne par semaine à l'intérieur du domicile ?

☐ Aucune ☐ Entre 1 et 5 ☐ Entre 5 et 20 ☐ Plus de 20

EI6 Au cours des 12 derniers mois, le domicile a-t-il été rénové ? Oui / Non

EI6bis Si Oui, quels produits / matériaux ont été utilisés pour la rénovation ? (Cochez toutes les cases utiles)

EI12.1. ☐ Du papier peint

EI12.2. ☐ De la chaux

EI12.3. ☐ Des peintures en phase aqueuse

EI12.4. ☐ Des peintures en phase solvant

EI12.5. ☐ Des panneaux de bois

EI12.6. ☐ Des panneaux en plâtre (type Placoplatre)

EI12.7. ☐ Des colles

EI12.8. ☐ Autres

Si Autres, précisez :
……………………………………………………………………………………………………
……………………………………………………………………………………………………
……………………………………………………………………………………………………

EI7 Au cours des 12 derniers mois, avez-vous équipé votre domicile de nouveaux meubles, électroménagers, tapis, rideaux, ou autres ? Oui/ Non

Si oui, cochez les éléments concernés :

- Matelas
- Rideaux
- Tapis
- Petit électroménager
- Gros électroménager
- Meubles
- Ordinateur
- Télévision
- Autres…………..

EI8 Y a-t-il de l'humidité ou des traces visibles de moisissures dans votre domicile?

☐ Non☐ Oui

Si oui, remplissez ce tableau :

**Il y a des tâches de moisissures grandes… :**	**Dans combien de pièce.s de votre domicile ?**
o …de moins de 0,3 m^2	Dans …….. pièces
o …entre 0,3 et 3 m^2	Dans …….. pièces
o …de plus de 3 m^2	Dans …….. pièces

EI9 A votre domicile, les revêtements de sols sont en (plusieurs réponses possibles)

- Parquet, bois
- Plastique (vinyle, PVC)
- Linoléum
- Carrelage, dalles en céramique
- Autre : ……………….

EI10 Concernant le port de chaussures chez vous, votre habitude est de :

- Retirer vos chaussures dès que vous entrez dans le domicile
- Retirer vos chaussures de temps en temps
- Porter la plupart de temps les mêmes chaussures que vous portez à l'extérieur

EI11 A quelle fréquence diriez-vous que vous utilisez (ou brûlez) dans le domicile … ?

	Jamais	1 à 5 fois par an	6 à 11 fois par an	1 à 3 fois par mois	Plusieurs fois par semaine	Tous les jours
**Des bâtons ou cônes d'encens**	○	○	○	○	○	○
**Des désodorisants d'ambiance**	○	○	○	○	○	○
**De la colle, du vernis, des peintures ou des solvants**	○	○	○	○	○	○
**Des bougies (hors cires naturelles)**	○	○	○	○	○	○
**Diffuseur d'huiles essentielles**	○	○	○	○	○	○
**Autre: ………………..**	○	○	○	○	○	○

EI12 Utilisez-vous, <u>quand c'est la saison</u>, des moyens de lutte (à diffusion) contre les insectes (ex : aérosols, plaquettes à mettre dans les prises, …) ? Ne tenez pas compte des pièges collants.

- ☐ Non
- ☐ Oui, tous les jours
- ☐ Oui, plusieurs fois par semaine
- ☐ Oui, une fois par semaine
- ☐ Oui, plusieurs fois par mois
- ☐ Oui, une fois par mois ou moins

EI13 Le domicile est-il équipé d'un garage attenant et communiquant et utilisé pour garer une/des voiture.s?

- ☐ Oui
- ☐ Non

EI14 Pour le nettoyage de votre domicile, vous utilisez (plusieurs réponses possibles) :

- o Des produits manufacturés labellisés écologiques ou biodégradables
- o Des produits manufacturés (achetés tels quels en magasin)
- o Des produits faits maison (principalement à base de bicarbonate de soude, vinaigre, savon noir, etc…)
- o Des huiles et cires (pour parquets)
- o Du savon inodore et de l'eau
- o Autre (s) : …………………………………………………………

EI15 En moyenne, combien d'heures par jour aérez-vous votre domicile:

En automne :	……. fois ……. min/jour	En hiver :	……. fois ……. min/jour
Au printemps :	……. fois ……. min/jour	En été :	……. fois ……. min/jour

EI16 Le domicile est-il équipé d'une ventilation mécanique contrôlée ?

- ☐ Oui
- ☐ Non

EI17 Votre domicile est-il équipé de :

	Oui ou Non?	Si oui, fréquence d'utilisation
Bornes wifi, box internet	Oui / Non	NB : Ici, fréquence de connexion à internet ou à la télévision via la borne wifi :<br><br>☐ Tous les jours<br>☐ Plusieurs fois par semaine<br>☐ Une fois par semaine<br>☐ Plusieurs fois par mois<br>☐ Une fois par mois ou moins
Connexion internet filaire / câbles Ethernet	Oui / Non	☐ Tous les jours<br>☐ Plusieurs fois par semaine<br>☐ Une fois par semaine<br>☐ Plusieurs fois par mois<br>☐ Une fois par mois ou moins
Téléphones fixes sans fil (DECT)	Oui / Non	☐ Tous les jours<br>☐ Plusieurs fois par semaine<br>☐ Une fois par semaine<br>☐ Plusieurs fois par mois<br>☐ Une fois par mois ou moins
Ordinateur(s) portable(s) ou tablette(s)	Oui / Non	☐ Tous les jours<br>☐ Plusieurs fois par semaine<br>☐ Une fois par semaine<br>☐ Plusieurs fois par mois<br>☐ Une fois par mois ou moins
Ordinateur(s) fixe(s)	Oui / Non<br>Si oui, combien ?	☐ Tous les jours<br>☐ Plusieurs fois par semaine<br>☐ Une fois par semaine<br>☐ Plusieurs fois par mois<br>☐ Une fois par mois ou moins
Cuisinière électrique classique	Oui / Non	☐ Tous les jours<br>☐ Plusieurs fois par semaine<br>☐ Une fois par semaine<br>☐ Plusieurs fois par mois<br>☐ Une fois par mois ou moins
Cuisinière électrique à induction	Oui / Non	☐ Tous les jours<br>☐ Plusieurs fois par semaine<br>☐ Une fois par semaine<br>☐ Plusieurs fois par mois<br>☐ Une fois par mois ou moins
Appareils électriques (sèche-cheveux, grille-pain, rasoir électrique, machine à café, …)	Oui / Non	☐ Tous les jours<br>☐ Plusieurs fois par semaine<br>☐ Une fois par semaine<br>☐ Plusieurs fois par mois<br>☐ Une fois par mois ou moins
Outils électriques (perceuses, visseuse, ponceuse, …)	Oui / Non	☐ Tous les jours<br>☐ Plusieurs fois par semaine<br>☐ Une fois par semaine<br>☐ Plusieurs fois par mois<br>☐ Une fois par mois ou moins
Télévisons	Oui/Non	☐ Tous les jours<br>☐ Plusieurs fois par semaine<br>☐ Une fois par semaine<br>☐ Plusieurs fois par mois<br>☐ Une fois par mois ou moins

ENVIRONNEMENT EXTÉRIEUR

EE1. <u>A votre connaissance</u>, retrouve-t-on l'une ou l'autre des situations suivantes à proximité de votre domicile ?

	Très proche (- 20m)	Proche (20-100m)	A distance moyenne (100-500m)	Loin (500m – 1km)	Très loin (+de 1km)		Je ne sais pas
Route à grand trafic	○	○	○	○	○		○
Société pharmaceutique / chimique	○	○	○	○	○		○
Atelier utilisant des solvants (ex: magasin de bricolage, atelier de peinture, …)	○	○	○	○	○		○
Friches industrielles/Terres contaminées	○	○	○	○	○		○
Lignes à haute tension aériennes	○	○	○	○	○		○
Câbles électriques souterrains	○	○	○	○	○		○
Transformateurs électriques	○	○	○	○	○		○
Antennes GSM	○	○	○	○	○		○
Voies de chemin de fer/tram/métro	○	○	○	○	○		○
Éoliennes	○	○	○	○	○		○
Installation d'incinération des déchets	○	○	○	○	○		○
Décharge/ Centre de compost/ Centre de tri	○	○	○	○	○		○
Pompe à essence	○	○	○	○	○		○
Garage avec activités d'entretien et de réparation	○	○	○	○	○		○
Imprimerie	○	○	○	○	○		○
Nettoyage à sec	○	○	○	○	○		○
Salon de coiffure	○	○	○	○	○		○
Autre.s: …………………………… ………..	○	○	○	○	○		○

FIN DU QUESTIONNAIRE – partie enquêteurs

A remplir par les enquêteurs :

Code de pseudonymisation ☐

EMDEX ☐

AIRBEAM ☐

Téléphone ☐

Equipe d'enquêteurs ☐

Merci de votre participation !
