# Supplementary material for: Assessment of Individual Exposure to Multiple Pollutants (Noise, Particulate Matter, and Extremely Low-Frequency Magnetic Fields) Related to Daily Life Microenvironments in the Brussels Capital Region: Protocol for a Cross-Sectional Study
Source: JMIR Res Protoc. 2025 Jul 3;14:e69407. doi: 10.2196/69407 (PMC12271967; doi:10.2196/69407)
Supplement: Multimedia Appendix 5 [file resprot_v14i1e69407_app5.pdf]

This document is an English translation of the ExpoHealth study questionnaire. During the study, the questionnaire was administered either in French or in Dutch, two of the national languages.

ExpoHealth-1 Study

Assessment of exposure to 50 Hz magnetic fields, air pollutants, endocrine disruptors and noise in Brussels, and its relationship to quality of life, health and risk perception

Contact details of the research coordination team:

ULB - Erasme Campus

Center for Research in Environmental and Occupational Health

Prof. Catherine Bouland -catherine.bouland@ulb.ac.be - 02 555 40 27

Route de Lennik, 808 | 1070 Brussels

Investigators:

Agathe Salmon –agathe.salmon@ulb.be
Zineb Ennamsa – zineb.Ennamsa@ulb.be
Maryse Ledent - Maryse.Ledent@sciensano.be

Questionnaire to be returned to the investigator after 24 hours of measurements or to be sent to:

Agathe Salmon (A.2.134)
Building A (ESP, ULB) – CP 593
Route de Lennik, 808
1070 Anderlecht
agathe.salmon@ulb.ac.be

Today's date: (day, month, year) ……/……/ …….
Code (to be completed by the investigators) ………………

ULB complies with the General Data Protection Regulation (GDPR) and attaches great importance to the protection of your personal data. This means that your previously provided contact details, as well as the personal information you provide in this questionnaire, will be stored securely and will only be accessible by the project's research team for the purpose of carrying out the project. They will not be shared with third parties under any circumstances. Furthermore, a procedure has been put in place to ensure that the data you pre-complete online will remain anonymous and will not be accessed by the research team until your final consent to participate in the research has been obtained. If you decide not to participate, your personal data will be deleted.

Any questions regarding this research may be directed to *agathe.salmon@ulb.be*, doctoral student in charge of the project. Any questions about the protection of your data by the ULB can be sent to the Data Protection Officer:*rgpd@ulb.be*.

Please read the following questions carefully and answer them independently and as completely and accurately as possible. As the questionnaire is long (+-1 hour), feel free to take breaks and come back to it later if your attention wanes.

We warmly thank you for your interest and participation in this study.

INFORMED CONSENT

Questionnaire as part of the ExpoHealth study

This questionnaire is part of the ExpoHealth Study, which aims to assess exposure to 50 Hz magnetic fields, air pollutants, endocrine disruptors, and noise in Brussels, and the relationship of these exposures to the quality of life, health, and risk perception. You are invited to pre-complete this questionnaire before our visit and the launch of the 24-hour measurements of your multiple exposures. Your participation in this questionnaire and the rest of the study is voluntary and must remain free of any coercion. You may stop your participation at any time and without justification.by informing the investigator (agathe.salmon@ulb.be).

(This question is required)
CE1. I declare to give my free and informed consent to participate in this questionnaire as part of the ExpoHealth study conducted by the *Center for Research in Environmental and Occupational Health at the ULB School of Public Health.* I understand that my participation in this questionnaire is voluntary and that I can stop it at any time without justification. I understand that my answers to this questionnaire will be pseudonymized and protected. The information provided will only be used for general statistics that may lead to scientific publications; but my individual data will not be made public.

I voluntarily agree to participate in this questionnaire.

- o Yes → Go to the next page
- o No → We thank you for your interest and invite you to inform the principal investigator that you no longer wish to participate in the Expo Health study by sending her an email to agathe.salmon@ulb.be .

PERSONAL DATA

DP1 You are: a man / a woman / non-binary ……….

DP2 What is your year of birth?

DP3 What is your nationality?………………..

DP4 You are (originately) from :

- o Europe
- o North Africa
- o Asia
- o North America
- o Central or South America
- o Sub-Saharan Africa
- o Middle East
- o Oceania

DP5 What is your highest level of education?

- o Non-graduate or holder of the basic studies certificate
- o Graduate of lower secondary education
- o Graduate of upper secondary education
- o Student or graduate of short-term higher education (bachelor)
- o Student or graduate of long-term higher education (master)
- o Doctoral student or graduate of 3rd degree of education (PhD)

DP6 What is your household made up of (people living at home)?

Number of adults: ……

Number of children: ……

DP7 In which bracket is the monthly taxable net income of your household ?

- o Below or equal to 1500 eur
- o Between 1500eur and 3500 eur
- o Between 3501eur and 5000 eur
- o More than 5000eur

DP8 If you live in a shared home/shared apartment, how many people living under the same roof depend on the income stipulated above?………

PROFESSIONAL INFORMATION

IP1 Professional status

Are you currently employed? Yes / No

If yes: In what proportion of the time do you work? ………%

Do you have a specific schedule (shift work, rotating hours, or night shifts)? Yes / No. If yes, what are your usual schedule(s)? ……………………………………..

If no: What is your current status?

- o Student
- o Housewife or man
- o Unemployed
- o Disability/illness/maternity, breastfeeding or parental leave
- o On career break
- o (Early) retired
- o Other:……………

IP3 In which sector(s) of activity have you worked in the last 10 years? Below are examples of the main sectors of activity. Please specify your function in each job.

EXAMPLES of sectors of activity :

- *Accommodation and catering*
- *Information and communication*
- *Scientific and technical activities*
- *Administrative service activities*
- *Education*
- *Human health*
- *Arts, entertainment and recreation*
- *Sport*
- *Sale*
- *IT Department*
- *Transportation and storage*
- *Construction*
- *Agriculture/Forestry/Fishing*
- *Manufacturing*
- *Production and distribution of electricity or gas*
- *Trade or repair of motor vehicles*
- *Unemployed or student*
- *Other: ………………*

Years	Sector of activity	Function
2020		
2019		
2018		
2017		
2016		
2015		
2014		
2013		
2012		
2011		
2010		

PLACE OF RESIDENCE

LR1 In what year did you move to this address? | | | | |

LR2 What type of residence is it?

- o Four-sided single-family house
- o Three-sided single-family house
- o Semi-detached single-family house
- o Apartment building
  - → What floor do you live on?....................
- o Other : ……………….

LR3 How many rooms are there in the accommodation?*(only count bedrooms and living spaces such as living rooms and dining rooms. Do not count the kitchen, bathroom, toilet, hallways, or storage rooms)*:……… living rooms.

ENVIRONMENTAL SENSITIVITY

SE1 Would you say that you are sensitive to the following environmental parameters?

	Not at all	Slightly sensitive	Moderately sensitive	Very sensitive	Extremely sensitive	I don't know
Noise from the neighbourhood						
Noise from the traffic						
Low and continuous noise (eg: neon lights, ventilation system, …)						
Electromagnetic fields generated by electrical devices						
Electromagnetic fields generated by the electrical network						
Electromagnetic fields generated by GSM antennas						
Exhaust gases from cars						
Poor air quality in general						
Pesticides in the food						
Others:…………..						
………………………						

PERCEPTION OF HEALTH AND SYMPTOMS

PS1 Overall, how is your health:

- ○ Very good
- ○ Good
- ○ Average
- ○ Bad
- ○ Very bad

PS2 During the past four weeks, how much have the following problems bothered you?

Physical symptoms

**Physical symptoms**	**Not at all**	**A little**	**A lot**
Stomach pain	o	o	o
Back pain	o	o	o
Pain in limbs or joints	o	o	o
Menstrual pain or other period-related problems (if applicable)	o	o	o
Pain or problems during sexual intercourse	o	o	o
Migraines	o	o	o
Chest pain	o	o	o
Dizziness	o	o	o
Syncope, fainting	o	o	o
Palpitations	o	o	o
Shortness of breath	o	o	o
Constipation, loose stools, diarrhea	o	o	o
Nausea, flatulence, or indigestion	o	o	o
Tinnitus	o	o	o

PS3 Over the past two weeks, how often have you experienced the following problems? Stress symptoms

**Stress symptoms**	**Never**	**Several days**	**More than half of time**	**Almost every day**
A feeling of nervousness, anxiety, or tension	o	o	o	o
An inability to stop worrying or to control worrying	o	o	o	o
Excessive worry about certain things	o	o	o	o
Difficulty relaxing	o	o	o	o
So much agitation that it is difficult to stay still	o	o	o	o
A tendency to be easily upset or irritable	o	o	o	o
A feeling of fear as if something terrible might happen	o	o	o	o

PS4 Over the past two weeks, how often have you experienced the following problems? Mood symptoms

**Mood symptoms**	**Never**	**Several days**	**More than half of the time**	**Almost every day**
Little interest or pleasure in doing things	o	o	o	o
Feeling sad, depressed, or hopeless	o	o	o	o
Difficulty falling or staying asleep, or excessive sleeping	o	o	o	o
Feeling tired or having low energy	o	o	o	o
Having little appetite or eating too much	o	o	o	o
Having a poor self-image – thinking that you are a failure, that you have disappointed your expectations or those of your family.	o	o	o	o
Difficulty concentrating on things such as reading the newspaper or watching television	o	o	o	o
Moving or speaking so slowly that other people might notice. Or, conversely, being so agitated or restless that you move much more than usual.	o	o	o	o
Thinking you would be better off dead or thinking about hurting yourself in some way.	o	o	o	o

PS5 Over the past two weeks, how often have you experienced the following issues?

	Never	Several days	More than half of the time	Almost every day
Difficulty concentrating	o	o	o	o
Memory problems	o	o	o	o
Difficulty finding words	o	o	o	o

PERCEPTION OF THE ENVIRONMENT

PE1 Evaluate the extent to which you feel concerned by the impact of the following agents on health, whether you are exposed to them or not.

	Not at all	A little concerned	Moderately concerned	Very concerned	Extremely concerned
Poor ventilation of buildings	o	o	o	o	o
Contamination in the water supply	o	o	o	o	o
Vaccination programs	o	o	o	o	o
Overuse of antibiotics	o	o	o	o	o
Toxic chemicals in household products	o	o	o	o	o
Radiation leaks from microwave ovens	o	o	o	o	o
Bacteria in air conditioning systems	o	o	o	o	o
Drug-resistant bacteria	o	o	o	o	o
Amalgams for dental fillings	o	o	o	o	o
Medical and dental x-rays	o	o	o	o	o
Air pollution	o	o	o	o	o
Noise pollution	o	o	o	o	o
Ozone layer depletion	o	o	o	o	o
Exhaust gas	o	o	o	o	o
SARS-CoV-2 virus (Covid-19)	o	o	o	o	o
Pesticide sprayer	o	o	o	o	o
Genetically modified foods	o	o	o	o	o
Additives in food	o	o	o	o	o
Pesticides in food	o	o	o	o	o
Antibiotics in food	o	o	o	o	o
Hormones in food	o	o	o	o	o
Cell phones	o	o	o	o	o
Radiocommunication or mobile telephone antennas	o	o	o	o	o
High voltage lines	o	o	o	o	o
Climate change/greenhouse gases	o	o	o	o	o
Electromagnetic fields generated by electrical devices	o	o	o	o	o
Electromagnetic fields generated by the electrical network	o	o	o	o	o
Others : ……………	o	o	o	o	o

PE2 To what extent do you think you are exposed to the following environmental parameters?

	Not at all	Slightly	Moderately	Very	Extremely	I don't know
Noise from the neighbourhood						
Noise from the traffic						
Low and continuous noise (eg: neon lights, ventilation system, ...)						
Electromagnetic fields generated by electrical devices						
Electromagnetic fields generated by the electrical network						
Electromagnetic fields generated by GSM antennas						
Exhaust gases from cars						
Pesticides in the food						

PE3 How often have you heard about the health risks of the following environmental parameters,<u>in the last 3 months</u> ?(All types of communication: for example, through friends, the media, on the street, etc.<u>except through this study</u>)

	Never (out of 3 last months)	1 to 2 times	More than 3 times
The noise	o	o	o
Electromagnetic fields generated by the electrical network	o	o	o
Electromagnetic fields generated by electrical devices	o	o	o
Electromagnetic fields from relay antennas (GSM)	o	o	o
Endocrine disrupting chemicals	o	o	o
Air pollution	o	o	o

PE4 Do you find it noisy in your home?*(If the noise is variable, give an average value)*

In my home, I find that it :

1 2 3 4 5

Very quiet *Very noisy*

- If you find the internal environment of your house/apartment noisy, explain why if possible:

  ……………………………………………………………………………………………………
  ……………………………………………………………………………………………………
  ……………………………………………………………………………………………………

PE5 How do you perceive the <u>indoor air quality in your home</u> ? *(If indoor air quality varies, give an average value)*

☐I find the air quality in my home to be:

1 2 3 4 5

Very good *Very bad*

- If you think the indoor air quality is poor, explain why if possible:

  ……………………………………………………………………………………………………
  ……………………………………………………………………………………………………
  ……………………………………………………………………………………………………

PE6 How do you perceive the <u>outdoor air quality around your home</u> ?*(If outdoor air quality varies, give an average value)*

☐I find the air quality around my home to be:

1 2 3 4 5

Very good *Very bad*

- If you think the outdoor air quality is poor, explain why if possible:

  ……………………………………………………………………………………………………
  ……………………………………………………………………………………………………

PE7 Do you think you are exposed to electromagnetic fields inside your home ?*(If exposure varies, give an average value)*

☐Regarding exposure to electromagnetic fields in my home, I believe I am

1 2 3 4 5

Not exposed at all *Very highly exposed*

- If you think your exposure is high, explain why if possible:

……………………………………………………………………………………….

…………………………………………………………………………………………………

PE8 Do you think you are exposed to electromagnetic fields in your neighborhood ?

(If outdoor exposure varies, give an average value)

☐Regarding exposure to electromagnetic fields in my neighborhood, I think I am:

1 2 3 4 5

Not exposed at all *Very highly exposed*

- If you think your exposure is high, explain why if possible:

………

………

GREEN SPACES: PERCEPTION AND ATTENDANCE

EV1 Does your home have an outdoor space?

- o Yes, a garden.
- o Yes a shared garden
- o Yes a terrace
- o Yes, a balcony.
- o No

EV2 Are there any green spaces in your neighborhood? [If you tick "not at all," skip to question EV4]

1 2 3 4 5

Not at all *A lot*

EV3 Do you visit the green spaces in your neighborhood?

1 2 3 4 5

Never *Very often*

EV4 Are you satisfied with the number of green spaces in your neighborhood?

1 2 3 4 5

Not at all satisfied *Totally satisfied*

EV5 Generally speaking, would you describe the streets in your neighborhood as "vegetated" (presence of flowerbeds, trees, bushes, grasses, etc.)?

YES/NO

EV6 Do you change your route when traveling on foot/by bike to go through these types of streets (flowery, green, airy)?

1 2 3 4 5

Never *Very often*

MEDICAL AND GENETIC HISTORY

MG1 What is your weight?………. Kg

MG2 What is your height?………cm

MG3 What is your usual blood pressure (if you know it)?…………/…………

MG4 Do you suffer or have you suffered from the following illnesses? If yes, complete the table below

**Pathology**	**Yes/No**	**From (month-year)**	**To (month-year)**	**Treatment followed**	**Details of the pathology (eg: *breast* cancer)**
Cancer					
Hepatitis					
Mononucleosis					
Herpes					
AIDS					
Meningitis					
Bacterial or viral infection (if recent)					
Cardiovascular disease					

Diabetes					
Respiratory allergy					
Concentration disorder					
Other:					

MG5 List any diagnostic or therapeutic x-rays other than dental that you have received in the past 5 years.Think X-rays and CT scans, not ultrasounds or MRIs.

Reason(s) for X-rays	Date(s) of X-rays (mm/yyyy)

MG6 Have you had a dental x-ray?Yes/No

If yes, check the period:

- o The previous month
- o In the previous 6 months
- o Within 6 to 12 months
- o More than a year ago

MG7 Are you aware of any birth defects or other genetic disorders or hereditary diseases that affect members of your biological family?

Yes / No

If yes, which family member(s)? Check the person(s) concerned and specify the disorder.

Biological family member	Nature of the disorder
oMother	………………………………………………
oFather	………………………………………………
oSister	………………………………………………
oBrother	………………………………………………
oChild	………………………………………………
oGrandfather	………………………………………………
oGrandmother	………………………………………………
oNephew or niece	………………………………………………
oUncle or aunt	………………………………………………

MG8 Concerning hereditary diseases:

- o I am a carrier of a hereditary disease (recessive gene)
  - Which ? …………………
- o I suffer from a hereditary disease
  - Which ? …………………
- o To my knowledge, I am neither a carrier nor affected by a hereditary disease.

MG9 Have you had difficulty conceiving a child (period of at least 12 months of inconclusive trying)?

- o Yes
  - If yes, indicate when you encountered this difficulty: From ...…/……… to ……/……… (month/year)
- o No
- o Not concerned (i.e., "didn't try")
  - *If you checked "no" or "not applicable", go to question MG13*

MG10 Have you ever had a fertility test (hormone test, spermogram, pelvic ultrasound, etc.)?Yes / No

MG11 Have you been diagnosed as infertile? Yes / No

If yes, indicate when you received the diagnosis: …/…… (month/year)

MG12 Are you taking hormonal medication?

- o Yes :
  - If yes, please specify:
    - o Hormone substitutes
    - o Contraceptive pill
    - o IUD or hormonal ring Other:
    - o …………….
- o No

LIFESTYLE AND BEHAVIORS

Smoking

LS1 Have you ever smoked (more than a year)?

Yes / No [If No, go to question LS5]

If Yes, how old were you when you started smoking? ....

LS2 Do you currently smoke? Yes / No

If no, how old were you when you quit smoking? ....

LS3 How many cigarettes did you smoke or do you smoke on average per day?

- o Less than one
- o Between 1 and 5
- o Between 5 and 20
- o More than 20

LS4 Do you smoke anything other than cigarettes (e.g. water pipe, etc.)?

- o Yes
  - If yes, what do you smoke? ……………….
  - How often do you smoke? …………………..
- o No

Drugs

LS5 Do you use or have you ever used recreational substances?

- o Yes / No
- o If you are currently using any, which one(s) are they?
  - o Cannabis,
  - o Hallucinogenic mushrooms,
  - o Ecstasy/MDMA,
  - o Cocaine,
  - o LSD,
  - o Amphetamine,
  - o GHB,
  - o Ketamine,
  - o Popper,
  - o Others) : ………………)

- O If you currently use it, how often do you do so?
  - o Several times a week
  - o Between 1 and 5 times a month
  - o Between 5 and 11 times a year
  - o Less than 5 times a year

Diet (current habits)

LS6 Complete the table below which shows your food consumption over one year.

<u>How to fill in the table:</u>

All questions are on your 12-month average consumption.

Fill in as follows:

Ex. 1: You never eat shellfish - check the "never" box

Ex. 2: You eat barbecued meat, but only in summer, about twice a week - Calculated for the whole year, this comes to an average of 8 to 10 times a year, so less than once a month

Ex. 3: You drink tea every 2 days, that is an average of 2-4 times a week

	AVERAGE CONSUMPTION DURING THE LAST 12 MONTHS								
	Never	times per month		times per week			times per day		
		less than 1	1 to 3	1	2 to 4	5 to 6	1	2 to 3	More than 3
Molluscs and crustaceans	x								
Meat and barbecue		x							
Tea					x				

Your food frequency table :

AVERAGE CONSUMPTION DURING THE LAST 12 MONTHS									
	Never	times per month		times per week			times per day		
		less than 1	1 to 3	1	2 to 4	5 to 6	1	2 to 3	More than 3
MEAT									
Red meat (ag: beef, mutton, lamb, etc.)									
White meat (eg: veal, poultry, rabbit, pork, etc.)									
Processed meat preparations (eg: sausages, hamburgers, nuggets, etc).									
Any meat cooked on the barbecue									
FRESH FISH AND SEAFOOD									
White fish (eg: cod, pollock, sea bass, sea bream, etc.)									
Oily fisheg (eg: salmon, tuna, herring, turbot, etc.)									
Molluscs and crustaceans (eg: mussels, octopus, clams, scampi, etc.)									
CANNED FISH									
Canned fish or seafood (eg: tuna, mackerel, anchovies, salmon, sardines, etc.)									
DRINKS									
coffee/tea									
softs									
herbal tea									
beer									
wine									
other alcohols									
WATER									
tap water (unfiltered)									
tap water (filtered)									
plastic bottled water									
SOY PRODUCTS									
Soy products (eg: tofu, soy milk, bean sprouts, soy sauce, etc.)									
DAIRY PRODUCTS									
whole milk									
full-fat yogurt									
hard cheese									
soft cheese									
cottage cheese									
butter									
Others:...........									
READY-MADE MEALS									
ready-made meat									
restaurants meals									

SWEETENING PRODUCTS				
artificial sweetener (eg: aspartame)				
CONTAINERS				
canned drinks				
canned food (eg: soups, ravioli, tomato sauce, ...)				

**This food frequency table is inspired by the Sciensano questionnaire "Belgian Food Consumption Survey 2014"*

LS7 Do you follow a specific diet?

- o Yes
  - If yes, which one:
    - o High protein
    - o Vegetarian
    - o Vegan
    - o Low in fat
    - o Low carb
    - o Other : ……………………
- o No

LS8 If you consume tea/herbal tea, in what form do you infuse it? (Multiple answers possible)

- o In a plastic or nylon container
- o In a paper container
- o In a metal container
- o Loose in hot water
- o Other:…………………
- o I don't drink tea or herbal tea.

LS9 Over the past month,<u>on all food consumed</u> , approximately what percentage was of organic food?…….%

LS10 In the last month, <u>on all food consumed</u> , approximately what percentage was wrapped in plastic?…….%

LS11 In the last month, <u>on all food consumed</u> , approximately what percentage was packaged in printed cartons?………%

LS12 If you consume organic products, where do you get them?

- Large stores (such as Carrefour, Delhaize, Lidl, Colruyt, Aldi, etc.)
- Major organic brands (Bioplanet, Carrefour bio, etc.)
- Organic neighborhood shop or grocery store
- Local producers, GASAP, organic baskets, etc.
- I don't eat organic food

LS13 If you use a water bottle several times a week,

- What material is it made of?
  - o It is plastic
  - o It is metal/stainless steel
  - o It is glass
  - o Other (specify): ………………
  - o I don't use a water bottle, or only occasionally [go to question LS14]

- What kind of drinks do you carry in it?
    - Hot
    - Cold
    - Both

LS14 Your kitchen utensils are in (multiple answers possible):

- Stainless steel
- Plastic/silicone
- Bamboo
- Other (specify): ……………….

LS15 Do you ever heat food in a plastic container (Tupperware, etc.) in the microwave?

- Yes, several times a week.
- Yes, a few times a month.
- Yes, but very rarely.
- No, never

Cosmetics

LS16What frequency best suits your use of the following products?

	**Every day**	**2-3 days a week**	**1 day a week**	**1 to 3 times per month**	**Less than 1 time per month**	**Never**
Soap						
Shampoo						
Conditioner						
Moisturizing product						
Day cream						
Night cream						
Shaving products						
Aftershave						
Hairspray						
Hair oil						
Tonic lotion						
Make-up						
Lip balm						
Deodorant						
Scent						
Nail polish						
Others: …………….						

LS17 You put on sunscreen:

- o Never
- o Only in summer (i.e., during periods of sun exposure)
- o Very often
- o Other : …….

LS18 Of all your cosmetic and care products, how many are:

	Homemade(with natural products)	Certified "organic"	Certified "without paraben"	Certified "without phthalates"	Certified "with antimicrobial action"	Without scent/perfume
None						
Less than 25%						
Half of them						
More than the half						
All of them						
I don't know						

GSM/Smartphones

LS19 Do you use a GSM/Smartphone?

Yes/No

If yes, how many hours per week-day on average?

- Less than 1 hour
- Between 1 and 3 hours
- Between 3 and 5 hours
- More than 5 hours

How many hours per weekend-day on average?

- Less than 1 hour
- Between 1 and 3 hours
- Between 3 and 5 hours
- More than 5 hours

LS20 How do you use your GSM/Smartphone?

Sending and reading SMS messages	Yes/No	Number per week on average:<br>○ 0-10<br>○ 11-50<br>○ 51-100<br>○ More than 100
Having a conversation on the phone	Yes/No	Average number of hours per week:<br>○ Less than 1 hour<br>○ 1 to 5 hours<br>○ 5 to 10 hours<br>○ More than 10 hours
Playing games and following social networks	Yes/No	Average number of hours per week:<br>○ Less than 1 hour<br>○ 1 to 5 hours<br>○ 5 to 10 hours<br>○ More than 10 hours
Listening to music and watching videos	Yes/No	Average number of hours per week:<br>○ Less than 1 hour<br>○ 1 to 5 hours<br>○ 5 to 10 hours<br>○ More than 10 hours
Using a calendar or your emails	Yes/No	Average number of hours per week:<br>○ Less than 1 hour<br>○ 1 to 5 hours<br>○ 5 to 10 hours<br>○ More than 10 hours
Others:	Yes/No	Average number of hours per week:<br>○ Less than 1 hour<br>○ 1 to 5 hours<br>○ 5 to 10 hours<br>○ More than 10 hours

LS20bis During telephone conversations, how do you hold your mobile phone/smartphone?

	Never	Sometimes	Often	Always
GSM/ smartphone to the ear				
Wireless headphones				
Wired headphones				
Speaker				

LS21 When you are not using your mobile phone, where do you usually keep it? (several answers possible)

	When you are awake	When you are asleep
In the backocket of your pants/skirt		
In the front pocket of your pants/skirt		
In your bag		
In your bra		
In a waist bag (belt type)		
It is placed elsewhere in the room where you are		
It is placed in different room than the one your are in		
Near your head (eg: under the pillow)		
Other:		

LS22 Over the past 7 days, approximately how many hours per day did you use earphones or headphones to listen to music/podcasts, etc. (Use excluding phone calls)

- o I didn't use headphones
- o I used headphones about ……h……min per day

Physical activities

Think about the physical activities you do at work, when you get around, and during your leisure time. What types of effort do you put in?

Intense physical effort

AP1 In the past 7 days, how often did you do vigorous physical activities? such as lifting heavy loads, fitness (indoor sports) or cycling briskly for at least 20 minutes?
……….. times

AP2 How much time did you spend on these intense physical efforts, on average? the days you practiced these physical activities?

- o . . . . minutes per day I did these activities
- o I don't know

Moderate physical effort

AP3 Over the past 7 days, how often have you done moderate physical activity? like carrying light loads, cycling at a steady pace, or playing doubles tennis for at least 20 minutes? Don't include walking!……. times

AP4 How much time did you spend on these moderate physical efforts, on average? the days you practiced these physical activities?

- o . . . . minutes per day I did these physical activities I
- o don't know

Walking

AP5 Consider the time you spent walking to work and home, walking to get from place to place, and for recreation and/or sports. In the last 7 days, how many times have you walked for at least 20 minutes? ……. times

AP6 How much time did you spend walking, on average? the days you walked?

- o . . . . minutes per day
- o I don't know

AP7 In general, you walk at:

- An intense rhythm that makes you breathe much harder than normal
- A moderate pace that makes you breathe a little harder than normal
- A slower pace where there is no change in your breathing
- I don't know how to answer

AP8 What best describes your leisure activities over the past year?(Only one answer possible!)

- Hard training and competitive sport more than once a week
- Jogging and other recreational sports or gardening, at least 4 hours per week
- Jogging and other recreational sports or gardening, no more than 4 hours per week
- Walking, cycling or other light activities at least 4 hours per week
- Walking, cycling or other light activities no more than 4 hours per week
- Reading, watching television or other sedentary activities
- I don't know how to answer

Travel

LS23 How do you usually travel to get to the following places:

	By foot	By bike	By bike or electric scooter	With the pubmic transport	By car or motorcycle	Mix of walk/bike+ public transport	Other
Work/school							
Your hobbies							
Shopping (groceries)							
Restaurant, movie, theatre,...							
Other:......							

LS24 If you make certain journeys on foot, by bicycle or by electric bicycle/ scooter, do you ever change your route to avoid:

Outdoor air pollution (perceived or actual):

- O Sometimes
- O Often
- O Always
- O Never

Traffic noise:

- O Sometimes
- O Often
- O Always
- O Never

LS25 If you make some journeys on foot, by bicycle or by electric bicycle/scooter: Is it generally easy to get around using these modes of transport in your neighborhood ? (Wide enough sidewalks, pedestrian zones, pedestrian and cyclist safety, cycle paths, ease of crossing major roads, etc.)

Yes/No

If not, what difficulties do you encounter?

……………………………………………………………………………………………

……………………………………………………………………………………………

……………………………………………………………………………………………

INDOOR ENVIRONMENT

EI1 If your home has an open fireplace, do you use it?

- o Yes
- o No
- o There is no open fireplace in the home

EI2 What is the main cooking energy you use?

- o Electric
- o Gas
- o Coal or wood stove

EI3 Is there an extractor hood in the kitchen?

- o Yes, and it is connected to the outside
- o Yes, but it is not connected to the outside
- o No

EI4 Is there gas heating in the home?

- o Yes and the air intake is connected to the outside
- o Yes and the air intake is not connected to the outside
- o No

EI5 How many cigarettes are smoked <u>on average per week</u> inside the home?

- ○ None
- ○ Between 1 and 5
- ○ Between 5 and 20
- ○ More than 20

EI6 <u>Over the past 12 months</u> , has your home been renovated? Yes / No

EI6bis If yes, what products/materials were used for the renovation? (Check all applicable boxes)

- ○ Wallpaper
- ○ Water-based paint
- ○ Solvent-based paint
- ○ Wood panels
- ○ Plasterboard panels
- ○ Glues
- ○ Others : ……………………

EI7 In the last 12 months, have you equipped your home with new furniture, appliances, carpets, curtains, or other items? Yes/No

If yes, check the relevant items:

- o Mattress
- o Curtains
- o Carpet
- o Small household appliances
- o Large household appliances
- o Furniture
- o Computer
- o Television
- o Others………….

EI8 Is there any dampness or visible signs of mold in your home?

☐ No ☐ Yes

If yes, fill in this table:

There are large mold stains…:	In how many rooms of your home?
o…less than 0.3 m2	In …….. rooms
o…between 0.3 and 3 m2	In …….. rooms
o…more than 3 m2	In …….. rooms

EI9 At your home, the floor coverings are in (multiple answers possible)

- o Parquet, wood
- o Plastic (vinyl, PVC)
- o Linoleum
- o Tiles, ceramic slabs
- o Other : ……………….

EI10 Regarding wearing shoes at home, your habit is to:

- o Remove your shoes as soon as you enter the house.
- o Take off your shoes from time to time
- o Wear the same shoes you wear outside most of the time

EI11 How often would you say you use (or burn) in the home…?

	Never	1 to 5 times per year	6 to 11 times per year	1 to 3 times per month	Several times per week	Every day
Sticks or incense cones	o	o	o	o	o	o
air fresheners	o	o	o	o	o	o
Glue, varnish, paintings or solvents	o	o	o	o	o	o
Candles (excluding natural waxes)	o	o	o	o	o	o
Oil diffuser essential	o	o	o	o	o	o
Other: ………………..	o	o	o	o	o	o

EI12 Do you use,<u>when it's the season</u> , means of fighting (by diffusion) insects (e.g. aerosols, tablets to put in sockets, etc.)? Ignore sticky traps.

- No
- Yes, every day
- Yes, several times a week
- Yes, once a week
- Yes, several times a month
- Yes, once a month or less

EI13 Is the home equipped with an adjoining and communicating garage used to park one or more cars?

- Yes
- No

EI14 To clean your home, you use (multiple answers possible):

- Manufactured products labeled ecological or biodegradable
- Manufactured products (purchased as is in store)
- Homemade products (mainly made from baking soda, vinegar, black soap, etc.)
- Oils and waxes (for parquet floors)
- Odorless soap and water
- Others) : …………………………………………………………….

EI15 On average, how many hours a day do you ventilate your home:

In autumn: ……. times ……. min/day In winter: ……. times ……. min/day

In spring: ……. times …….min/day In summer: ……. times ……. min/day

EI16 Is the home equipped with controlled mechanical ventilation?

- Yes
- No

EI17 Is your home equipped with ?

	Yes or No ?	If yes, what is the frequency of use ?
WIFI hotspots, ineternet boxes	Yes/No	o Every day<br>o Several times a week<br>o Once a week<br>o Several times a month<br>o Once a month or less
Wired internet connection (ethernet cables)	Yes/No	o Every day<br>o Several times a week<br>o Once a week<br>o Several times a month<br>o Once a month or less
DECT	Yes/No	o Every day<br>o Several times a week<br>o Once a week<br>o Several times a month<br>o Once a month or less
Laptops	Yes/No	o Every day<br>o Several times a week<br>o Once a week<br>o Several times a month<br>o Once a month or less
Desktop computer (fixed)	Yes/No	o Every day<br>o Several times a week<br>o Once a week<br>o Several times a month<br>o Once a month or less
Electric stove	Yes/No	o Every day<br>o Several times a week<br>o Once a week<br>o Several times a month<br>o Once a month or less
Induction cooker	Yes/No	o Every day<br>o Several times a week<br>o Once a week<br>o Several times a month<br>o Once a month or less
Electrical appliances (eg: hair dryer, toaster, coffee machine,...)	Yes/No	o Every day<br>o Several times a week<br>o Once a week<br>o Several times a month<br>o Once a month or less
Tools (eg: drills, screwdrivers,...	Yes/No	o Every day<br>o Several times a week<br>o Once a week<br>o Several times a month<br>o Once a month or less
Television	Yes/No	o Every day<br>o Several times a week<br>o Once a week<br>o Several times a month<br>o Once a month or less

OUTDOOR ENVIRONMENT

EE1.To your knowledge , do we find one or other of the following situations near your home ?

	Very close(- 20m)	Close (20-100m)	At a moderate distance (100-500m)	Far (500-1000m)	Very far (More than 1 km)	I don't know
Busy road						
Pharmaceutical company						
Fabric or store using solvents						
Industrial wastelands						
High voltage power lines						
Underground electric cables						
Electrical transformer						
GSM antennas						
Rail, tram or metro tracks						
Wind turbines						
Waste incinerator						
Landfill						
Gaz station						
Garage						
Printing company						
Dry cleaning						
Hair salon						
Other:.............						

END OF QUESTIONNAIRE – investigators section

To be completed by the investigators:

Pseudonymization code ☐

EMDEX ☐

AIRBEAM ☐

Phone ☐

Investigative team ☐

Thank you for your participation!
