# Supplementary material for: Assessment of Individual Exposure to Multiple Pollutants (Noise, Particulate Matter, and Extremely Low-Frequency Magnetic Fields) Related to Daily Life Microenvironments in the Brussels Capital Region: Protocol for a Cross-Sectional Study
Source: JMIR Res Protoc. 2025 Jul 3;14:e69407. doi: 10.2196/69407 (PMC12271967; doi:10.2196/69407)
Supplement: Multimedia Appendix 9 [file resprot_v14i1e69407_app9.pdf]

# Study Expo Health-1: Procedure for participants

You have agreed to take part in the ExpoHealth-1 study. Your participation will involve wearing measuring devices for 24 hours, answering a questionnaire (prior to the 24-hour measurement period) and recording the places where you are in 15-minute intervals during the 24-hour measurement period.

Prior to our meeting,

1) You will receive an invitation to choose the date that suits you best for starting the measurements. We estimate that the meeting will take about 15 minutes to answer your questions and provide you with the equipment.
2) Once you have selected the date, you will receive a new email with :
   a. **The information and consent document. We ask you to read it carefully and prepare any questions you may have before our visit.** We will sign it together only after we have answered all your questions about the study and if you wish to take part.
   b. A link to the questionnaire (or a paper version if requested) to be completed before our meeting. Please allow between 1 and 1.5 hours for the questionnaire to be completed.
   c. A video explaining how to take measurements, to be watched before and during the 24-hour measurement period, if required.

The day we meet (+-15min) :

NB: In order to comply as closely as possible with health measures against Covid-19, we will always wear a mask, disinfect our hands and all equipment, and maintain a distance of 1.5m from you. The meeting can take place on the doorstep, so as to remain outdoors, and will last around 15 minutes.

1) We will take the time to answer any questions you may have about the study, and together we will sign the Information and Consent Form (ICF).
2) Only after signing the ICF will we provide you with activated and disinfected portable devices. You will be required to wear them for 24 hours.
3) Finally, we will show you how to fill in the diary of microenvironments

The next day (10-15min) :

The next day, we will be back in the same time slot to collect the equipment and the completed diary of microenvironments.

# Description of equipment and procedure

## 1. Magnetic field measurement (Emdex)

The device is carried in the black shoulder pouch. Don't touch it for 24 hours, just carry it with you at all times during the day and when you're out and about. When you're asleep or when you can't keep it with you, place it next to you (less than 1m away if the phone is also in the pouch).

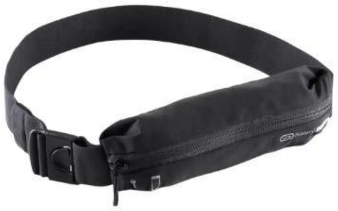

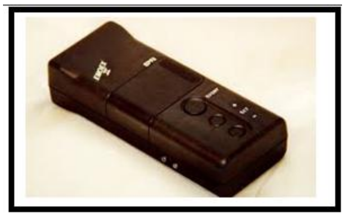

## 2. Measuring air quality and noise pollution (Airbeam2)

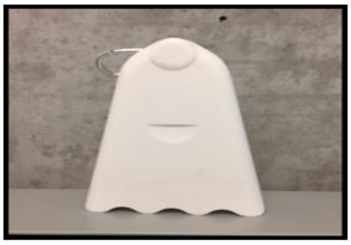

We'll activate it with you. It's ideal for wearing on your belt or shoulder strap. The data it collects is recorded on an application (phone supplied) via Bluetooth (Airbeam2 and phone must therefore always be no more than 1 metre apart to avoid disconnection). Airbeam2 has an 8-hour battery life, so you'll need to recharge it while you're sleeping and potentially during the day.

## IMPORTANT NOTES:

- Put **Airbeam2 to charge** next to you while you sleep (minimum 6 hours charge time).
  - Airbeam2 does not have a battery charge indicator. During charging, the indicator light is green. The device will remain on during the charge, so the green light will not go out after the 6-hour charge.
  - In the morning, check that the device is still working by listening to whether the pump is still working and hang the Airbeam2 on you for the rest of the day.
- Do not expose Airbeam2 directly to a plume of smoke (exhaust fumes, cigarettes, etc.) or mist (bathroom, etc.). As Airbeam2's measurement principle is based on optics, smoke particles would be deposited on the optical parts of the device, causing the measurement to drift.
- If possible, avoid vacuuming during the 24-hour measurement period.
- The **smartphone** in the pouch must remain **within 1 metre of Airbeam2 to maintain the Bluetooth connection**! Ideally, you should wear it on your belt or shoulder strap. Note that the smartphone does not contain a sim card; it is designed solely to store the data collected by AirBeam.
- Do not open the pouch to avoid interfering with the measurements taken by the Emdex and the phone.
- To avoid interference, **Airbeam2 must not be closer than 10 cm to the Emdex**.
- While Airbeam2 is operating, the **pump must be kept free of obstructions**.
- During the day, check that the device continues to measure by tilting your ear from time to time to ensure that the pump is still operating (-> if this is not the case, contact us).
- If the device stops measuring, the indicator light will flash red for a few seconds (-> contact us).

## 3. The diary of microenvironments

Take the time to fill it in several times a day. Simply note down the code corresponding to the type of environment you find yourself in, in 15-minute increments.

NB: Do not walk specifically towards sources of pollution with your devices, as this will confuse the data. We need to know your exposure levels on a normal day, as if you were not wearing the devices.

If you are in any doubt, or if Airbeam2 stops working, please contact us:


- Agathe Salmon [Study manager] : agathe.salmon@ulb.ac.be - 0470/08.13.62
- Zineb Ennamsa [Technician for the study] : zineb.ennamsa@ulb.ac.be - 02/555.40.28
